# Biosynthetic blood surrogates: Current status and future opportunities

**DOI:** 10.1002/btm2.70084

**Published:** 2025-10-24

**Authors:** Dante Disharoon, Sonali Rohiwal, Selvin Hernandez, Bipin Chakravarthy Paruchuri, Rohini Sekar, Norman Luc, Anirban Sen Gupta

**Affiliations:** ^1^ Department of Biomedical Engineering Case Western Reserve University Cleveland Ohio USA

**Keywords:** biomaterials, biosynthetic blood surrogate, blood transfusion, Global Health, nanoparticles

## Abstract

Blood is a liquid connective tissue containing cellular and non‐cellular components. Blood circulation is vital to life since it transports gases and nutrients, maintains immune surveillance, promotes necessary clotting to prevent hemorrhage, and maintains oncotic pressure and body temperature. Blood transfusion is a life‐saving procedure where donor‐derived blood is administered into a patient when the patient's own blood is diseased or depleted. However, blood transfusion faces tremendous challenges due to donor shortage, limited shelf life, transfusion‐associated infection risks, and complex logistics of blood banking and transport. A robust volume of research is currently focused on resolving these issues, including pathogen reduction technologies, temperature‐reduced storage, and bioreactor‐based production of blood cells from stem cells in vitro. In parallel, significant interest has developed toward biomaterials‐based engineering of synthetic blood surrogates that can provide critical functions of blood components while circumventing the limitations of donor‐derived blood products. Here, the major efforts have focused on the design of RBC surrogates for oxygen transport and platelet surrogates for hemostatic functions, and only limited efforts have focused on WBC mimicry. Processes have also been developed to isolate plasma or coagulation factors to treat specific bleeding risks, as well as freeze‐dry or spray‐dry plasma for long‐term storage and on‐demand use. The current article will provide a comprehensive review of various blood surrogate approaches highlighting biomaterials design and applications, important challenges, and future opportunities.

## Graphical abstract

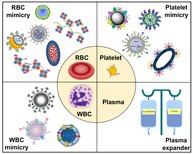

**Translational Impact Statement**

Blood transfusion is essential to save lives and support health in pregnancy‐related hemorrhage, traumatic injuries, surgeries, and inherited or acquired bleeding complications. However, currently, all blood products come from blood donors and there is a persistent shortage of donors. Furthermore, donated blood products face additional challenges of contamination risks, limited shelf‐life, and portability, and specialized storage. Biosynthetic blood surrogates can provide a lifesaving solution to address these critical challenges to preserve global health.

## 1 INTRODUCTION

**FIGURE 1:**
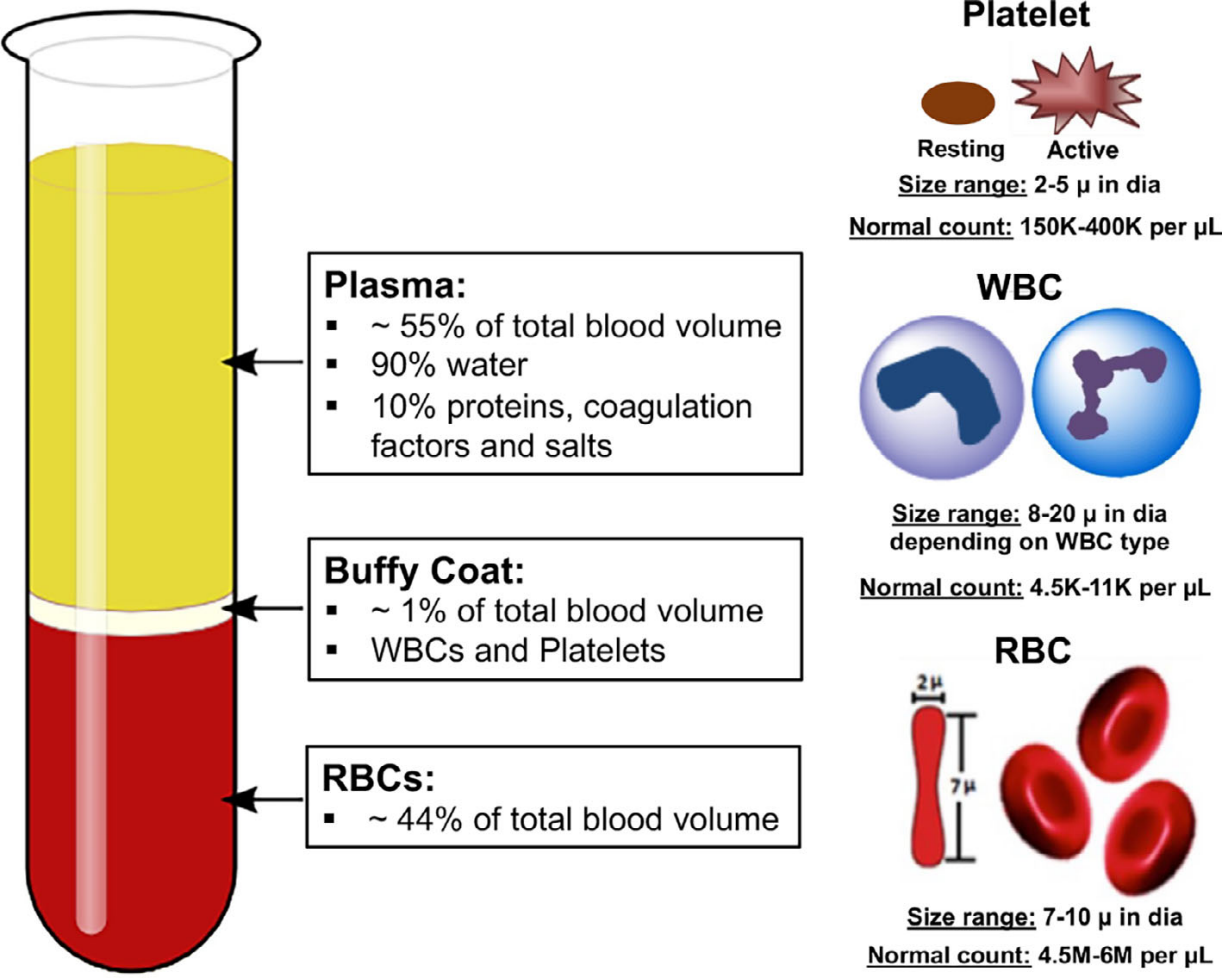
Schematic of cellular and molecular composition of blood along with normal cell counts and size ranges for the various blood cells.

Blood is considered a “liquid connective tissue” where living cells (e.g., red blood cells or RBCs, white blood cells or WBCs, and platelets), proteins (e.g., albumin, immunoglobulins, fibrinogen, coagulation factors, etc.), and salts (e.g., chloride and bicarbonate salts of sodium, potassium, calcium, magnesium, etc.) are suspended in an aqueous matrix. In humans, children have 70–90 mL per kilogram body weight of blood volume, while in adults there is approximately 5 L total blood volume. Of the cellular components, RBCs make up approximately 45% of the blood volume, while WBCs and platelets make up about 1%–2% of the blood volume. The rest of the blood volume is the aqueous plasma carrying the proteins and salts. Blood circulation is vital to life since it transports respiratory gases (e.g., O_2_ transport by RBCs and CO_2_ transport by RBCs and plasma), carries nutrients, maintains immune surveillance (e.g., by WBCs), promotes clotting to prevent blood loss (e.g., by platelets and coagulation factors), and maintains oncotic pressure and body temperature (e.g., by plasma). Figure 1 shows a schematic representation of blood composition along with the size and normal count information of the three blood cell types.^1^, ^2^, ^3^, ^4^

Since the description of blood circulation by William Harvey in the 17th century and the landmark discovery of blood groups by Karl Landsteiner in the 19th century, our understanding of blood physiology and functions has grown significantly.^5^, ^6^ Blood transfusion is a clinical approach where donor‐derived blood is transfused into a recipient patient when the patient's own blood supply is diseased, dysfunctional, or depleted. Centuries ago, when “blood donation” was not common, there were attempts to transfuse milk, wine, salt solutions, and so forth, as “lood substitutes,” all of which failed due to their functional inadequacy compared to natural blood. In the modern age, transfusion of donor blood products has become a clinical mainstay. Approaches for preserving and transporting donor‐derived blood were reported during World War I to treat battlefield injuries, and during World War II blood transfusions had become widely available. During the 1950s, multiple blood banks were established in the United States and blood donation was promoted as a form of civic responsibility. Currently, whole blood (WB) as well as isolated components are clinically approved for transfusion in civilian as well as battlefield settings, chronic and acute anaemias, and disease‐associated, drug‐induced, or congenital bleeding disorders.^7^, ^8^, ^9^, ^10^, ^11^, ^12^, ^13^, ^14^, ^15^ The US military has long established strategic and logistic measures for “walking blood banks” in the battlefield.^16^, ^17^, ^18^ With the development of blood fractionation technologies, civilian blood banks adapted component‐based transfusion approaches (e.g., isolated RBC transfusion, isolated platelet transfusion, isolated plasma transfusion, etc.). During the past and current decade, a significant number of clinical trials have established the survival benefit of donor‐derived WB or blood component transfusion.^19^, ^20^, ^21^, ^22^ However, according to the World Health Organization (WHO) and the American Red Cross, there are persistent donor shortages within the US and globally, more so during challenging time periods such as the current COVID‐19 pandemic.^23^, ^24^, ^25^ Furthermore, donor‐derived blood products have limited shelf‐life due to contamination risks. For example, fresh WB must be used within 24 h; refrigerated WB has a shelf‐life of ~35 days; refrigerated RBCs have a shelf‐life of ~40 days, while platelet suspensions usually stored at room temperature per FDA guidelines have a shelf‐life of ~5–7 days.^26^, ^27^, ^28^ Additionally, stored RBCs and platelets undergo morphological, functional, and metabolic changes (collectively termed “storage lesions”), which affect their stability, in vivo circulation lifetime, and relevant bioactivities.^29^, ^30^ Critical pathogen reduction technologies (PRT) like psoralen‐based or riboflavin‐based UV irradiation, extensive serological testing of donor blood, leukoreduction, and specialized storage protocols have been established to reduce contamination risks in donor blood.^31^, ^32^, ^33^ Additionally, significant research is currently being directed toward enhancing the shelf‐life of blood products by reduced temperature storage and processing (e.g., chilling, freezing, lyophilizing, etc.). A variety of approaches are also being studied regarding the refinement of the fluid composition of the storage medium as well as the material composition of the storage bags to potentially improve the shelf‐life of donor‐derived blood cells.^34^, ^35^, ^36^, ^37^, ^38^ While these approaches have partially improved the shelf‐life, safety, and availability logistics of blood products, it is nowhere near the optimum level. Additionally, donor‐derived (i.e., allogeneic) blood products continue to present risks of transfusion‐associated immune reactions, refractoriness, and acute lung injury.^39^, ^40^ Altogether, these challenges arising from donor shortages, limited shelf‐life, contamination risks, and infection/immune risks for blood products have generated significant interest in “donor‐independent” solutions.^41^

One area of this approach is the in vitro production of RBCs and platelets from stem cells, utilizing unique bioreactor designs and culture conditions (a field of research interestingly termed “blood *pharm‐*ing”).^42^, ^43^, ^44^ This approach utilizes pluripotent stem cells or immortalized progenitor cells, cultured in unique 2D and 3D bioreactor designs in the presence of specific cytokines and growth factors to induce differentiation and production of RBCs and platelets. The use of biomaterials in this approach is in the cell culture scaffolds as well as in the bioreactor design. For example, cell‐seeding scaffolds have used polyethylene terephthalate (PET) membranes, polyacrylamide‐based hydrogel systems enriched with fibronectin and thrombopoietin, silk‐based tubular vascular mimetic niche, poly(dimethylsiloxane) or PDMS‐based microfluidic “lab‐on‐a‐chip” designs, NANEX nanofibers, structurally graded collagen scaffolds, and so forth, and bioreactor designs have used polycarbonates, PDMS, and so forth.^45^, ^46^, ^47^, ^48^, ^49^, ^50^, ^51^, ^52^, ^53^, ^54^, ^55^ Several research groups have also focused on the effect of media perfusion rate, turbulence, and fluid dynamic shear forces on the platelet production efficacy.^56^, ^57^, ^58^ While these various approaches to donor‐independent in vitro blood cell production continue to evolve and generate exciting and elegant scientific data, significant translational challenges remain to be resolved.^59^, ^60^, ^61^, ^62^, ^63^, ^64^ The first challenge is the maintenance of the fidelity and reproducibility of the “source” cells as well as differentiated cells, regarding genetic integrity, cell marker characterization, and prevention of final anucleate “RBC and platelet products” from getting contaminated with nucleated source cells. The second challenge is the current lack of a standardization protocol regarding the quality and the functional potency of the bioreactor‐produced RBC and platelet products. Different characterization methods spanning flow cytometry‐based assessment of cell markers to specific cellular activity assays have been reported by various research groups, but a clinically translatable standardization metric is yet to be established. The third challenge is that of scalability of the RBC and platelet products to clinically relevant transfusion units. A therapeutic unit of donor‐derived RBC product contains ~2 × 10^12^ RBCs and a therapeutic unit of donor‐derived platelet product contains ~3 × 10^11^ platelets.^48^, ^61^ Robust bioreactor processes that can achieve this scale at GMP standards while maintaining reproducible genetic and functional fidelity of the product is a significant hurdle. The fourth challenge is that of the cost of production and optimization of the cost‐to‐benefit framework. Currently, one unit of donor‐derived RBCs costs $200–300 and one unit of donor‐derived platelets costs $250–500. In comparison, recent analyses of the various asynchronous steps of cell culture, scale‐up, isolation, purification, characterization, and so forth, that are involved in bioreactor‐based GMP quality RBC and platelet production estimated that the cost of RBC or platelet production could be in the $10,000–$150,000 per unit, which may become a significant financial barrier to clinical translation.^59^, ^64^ Therefore, a significant opportunity remains in designing and utilizing biomaterials‐based unique 3D bioreactor processes that can address these existing challenges of in vitro blood cell production.

An alternative (and potentially complementary) approach for achieving “donor‐independent RBC and platelet systems” that has emerged is the development of biomaterials‐based blood substitutes that *functionally mimic* the biological mechanisms of blood cells while allowing reproducible large‐scale in vitro manufacture, sterilization, and long‐term storage, universal applicability (no need for type matching), and widespread availability on‐demand.^65^, ^66^, ^67^, ^68^, ^69^, ^70^, ^71^ In fact, design approaches for “synthetic RBCs” were reported by Prof. Thomas Chang as early as in the 1950s, and the interest in synthetic blood substitutes further developed during the HIV crisis of the 1980s due to the fear of contaminated blood products.^66^, ^72^, ^73^, ^74^ Interestingly, a 2008 meta‐analysis of 16 clinical trials of five different artificial RBC products suggested increased health risks in patients treated with such RBC surrogate products.^75^ Although such analyses have resulted in some apprehension in the clinical utility of these products, it has also directed significant emphasis on understanding and resolving the problems posed by these products at materials design, mechanistic, and functional levels, using unique biomaterials approaches. The following sections will comprehensively review various biomaterials‐based approaches for synthetic blood surrogates, highlighting the current state of the art and critically discussing the potential challenges and opportunities in this area.

## 2 RBC SURROGATES AND OXYGEN CARRIER SYSTEMS

**FIGURE 2:**
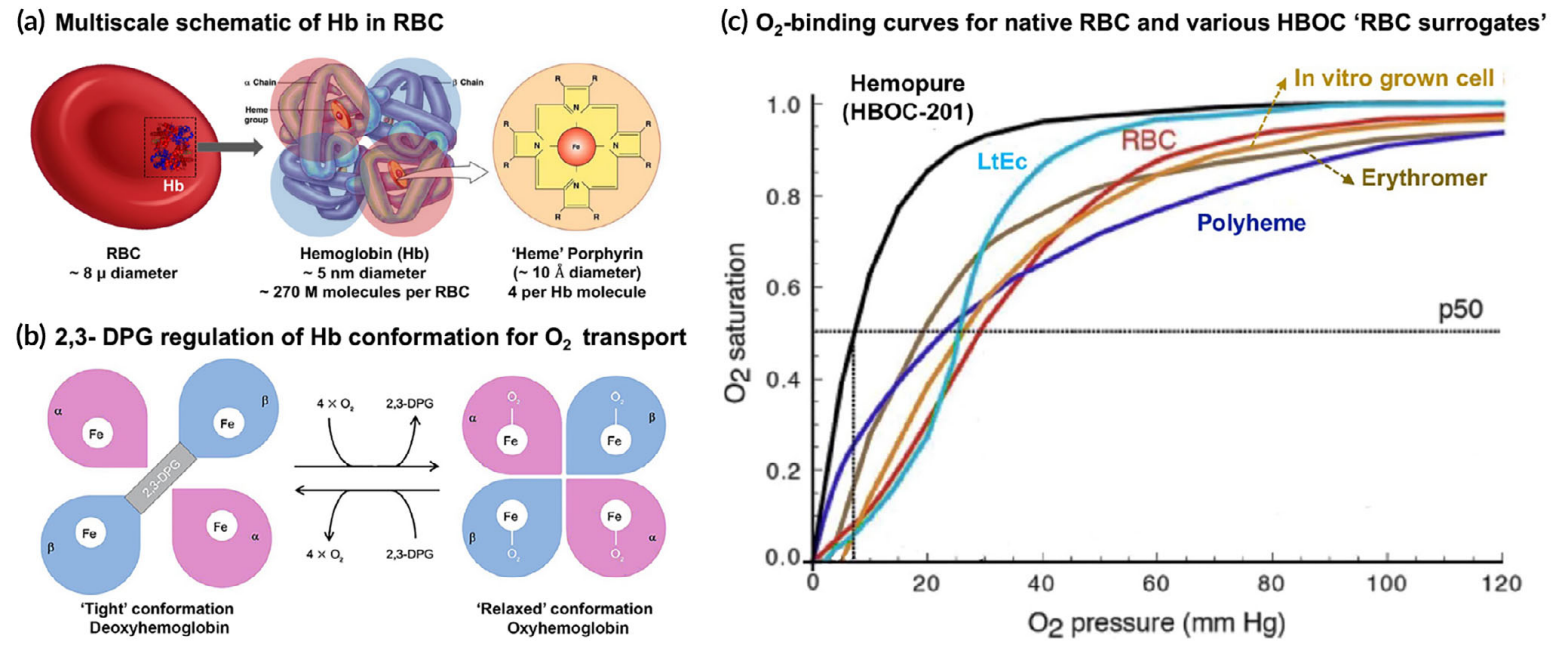
(a) Multiscale schematic of RBC and encapsulated hemoglobin within the RBC (Hb); (b) Schematic showing conformational changes in Hb regulated by 2,3‐DPG associated with binding and release of oxygen (O_2_); (c) Representative O_2_‐binding curves for Hb in its natural form (in RBC) versus for Hb incorporated in various “RBC surrogate” oxygen carrier technologies showing the sigmoid nature of the oxygen binding kinetics of Hb (adapted from Reference [97]).

RBCs primarily function in the blood to transport oxygen (O_2_) to tissues and partly transport carbon dioxide (CO_2_) from tissues, via binding of the gases to the protein hemoglobin (Hb) inside the RBCs. The average amount of Hb contained within adult human RBCs is ~30 pg per cell (~270 million Hb molecules), existing as a tetrameric protein of two *α*‐ and two *β*‐polypeptide units, each bearing an iron (Fe)‐containing “heme” group that can bind an O_2_ molecule.^76^, ^77^ The binding of O_2_ to Hb heme is positively cooperative, such that a small variation in oxygen partial pressure (pO_2_) causes a large change in O_2_ binding by Hb, as depicted by the characteristic sigmoidal O_2_‐binding curve.^78^, ^79^ The O_2_‐binding iron in Hb exists in its reduced “ferrous” (Fe^2+^) state, which upon binding O_2_ gets oxidized to the “ferric” (Fe^3+^) state. Therefore, in RBCs the O_2_‐binding mechanism of Hb is coupled to redox systems (e.g., cytochrome b5 reductase enzyme function), such that the Fe^2+^ oxidized to Fe^3+^ is cyclically reduced back to Fe^2+^ to enable cyclical O_2_‐binding and release by Hb. Additionally, inside RBCs the Hb undergoes conformational changes to allow higher O_2_ affinity in the lungs (higher O_2_ saturation) and lower O_2_ affinity in the tissue capillaries (desaturation for O_2_ release to tissues). Such cyclical conformational regulation of O_2_‐affinity in Hb is mediated by allosteric effector molecules like 2,3‐diphosphoglycerate (2,3‐DPG) formed inside RBCs as a glycolytic intermediate. Figure 2a shows the schematic structure of Hb molecule in RBCs and the iron‐containing “heme” pockets within Hb; Figure 2b shows the schematic of 2,3‐DPG regulated conformational change in Hb for O_2_ loading and release. Therefore, engineering of RBC surrogates poses complex challenges regarding thermodynamic and kinetic aspects of O_2_ transport by Hb.^80^, ^81^ From a biomaterials standpoint, the three important categories that are being developed for RBC surrogate technologies are: (1) Hb‐based oxygen carriers (HBOCs), perfluorocarbon (PFC)‐based systems, and iron (Fe^2+^)‐containing porphyrin systems.

### 2.1 Hemoglobin‐based oxygen carrier (HBOC) systems

HBOCs are RBC surrogates that utilize Hb from biological sources as the O_2_‐carrying component. Here, the Hb is used either directly or further modified chemically or encapsulated within “carrier particles” for protection and circulation stability. The Hb in HBOCs is sourced from human RBCs, bovine RBCs, annelid Hb, or from recombinant Hb manufacture.^82^, ^83^, ^84^ For isolating Hb from human or bovine RBCs, the cells are lysed, the Hb protein is isolated, purified, and sterilized to yield “cell‐free Hb” that has low antigenicity and retains oxygen‐binding capabilities. However, transfusion of such cell‐free Hb in patients showed extensive renal toxicity and cardiovascular complications.^85^, ^86^ It was found that cell‐free Hb has a very short circulation lifetime due to the rapid dissociation of the Hb tetramer into dimeric and monomeric forms, which can then bind to plasma immunoglobulins and undergo rapid clearance into the spleen, liver, and kidneys, causing substantial organ toxicity.^87^ Cell‐free Hb and its dissociated dimers and monomers also extravasate into the sub‐endothelial region and rapidly sequester nitric oxide (NO), converting NO into nitrate (dioxygenation) and oxy‐Hb to Met‐Hb.^88^, ^89^, ^90^ NO is a native vasodilator and therefore its sequestration by cell‐free Hb causes unwanted vasoconstrictions leading to cardiovascular issues. The iron‐containing “heme” of Hb also has inherent inflammatory and pro‐thrombotic effects toward vascular endothelium and platelets, thus causing additional thrombotic risks.^91^, ^92^ Also, since cell‐free Hb does not contain 2,3‐DPG, it suffers from the lack of conformational modulation of the O_2_‐affinity of Hb, leading to sub‐optimal tissue oxygenation. Altogether, these reasons have led to the failure of utilizing human or bovine RBC‐derived cell‐free Hb directly for RBC surrogate applications. To address these issues, some research has been directed toward exploring very high molecular weight Hb molecules found in earthworms (e.g., *Lumbricus terrestris* erythrocruorin or LtEc molecule) and marine worms (e.g., *Arenicola marina* Hb M101 molecule).^93^ However, in vivo safety of such marine Hb in preclinical mammalian models is yet to be established. Other research activities have focused on synthesizing Hb via recombinant technologies (e.g., in *Escherichia* coli) where specific mutations could allow reduced dissociation and NO‐binding capacities of Hb.^94^, ^95^, ^96^ However, the correct combination of mutations needed for an ideal recombinant Hb design remains unresolved. Therefore, the majority of approaches in utilizing human or bovine RBC‐derived Hb for RBC surrogates have focused on “biomaterials”‐based strategies, including chemical modification of Hb via cross‐linking, polymerization, macromolecular surface modifications, or Hb encapsulation within nanoparticle and microparticle carriers, to improve stabilization, circulation lifetime, safety, and function in vivo.^97^, ^98^, ^99^ Figure 2c shows O_2_‐binding curves for Hb in native RBC (red curve) compared to several HBOC “RBC surrogate” designs that have been extensively studied, as described in the next sections.

#### 2.1.1 Chemical modifications of Hb for HBOC applications

To reduce the dissociation of cell‐free Hb, a variety of direct chemical modifications of Hb have been investigated. One approach is the utilization of intra‐ and inter‐molecular crosslinking of Hb. For example, crosslinking of the *α*‐subunits using acylation with 2,2′‐[(1,4‐Dioxo‐1,4‐butanediyl)bis(oxy)]dibenzoic acid (also known as Diaspirin) resulted in the human Hb based HBOC product HemAssist (Baxter, USA).^100^, ^101^, ^102^ The circulation lifetime of HemAssist was found to be ~12 h compared to <6 h for non‐crosslinked cell‐free Hb; however, clinical trials with this product showed a 72% increase in mortality, leading to trial discontinuation. Similar crosslinking of the *α*‐subunits of recombinant Hb was done using glycine, leading to a product called Optro (Somatogen, USA), which also showed risks of cardiac arrest and mortality in clinical trials.^103^, ^104^ Instead of such *intra*molecular crosslinking, in another approach, Hb was polymerized via *inter*molecular crosslinking using glutaraldehyde or o‐raffinose. Examples of this approach are in the products Hemopure from Biopure, USA, PolyHeme from Northfield Labs, USA, and HemoLink from Hemosol, Canada.^105^, ^106^ While such polymerization of Hb reduces the rapid dissociation, a persistent issue is the lack of precise control over the crosslinking and polymerization extents, and rigorous purification steps become necessary to ensure product quality. PolyHeme did not exhibit major vasoconstrictive effects in safety studies and progressed into Phase III clinical trials in the United States in the 1990s in the Acute Normovolemic Hemodilution (ANH) setting, as well as in trials treating patients suffering from traumatic hemorrhagic shock under emergency research waiver and special protocol framework implemented by the FDA.^107^ However, after several fatalities occurred in these trials, the studies were canceled in 2001. Clinical trials with HemoPure (BioPure) showed favorable safety profiles and a reduced need for blood transfusions in cardiac surgery.^108^ This product is currently approved in South Africa for treating acute anemia but is yet to be approved in the US by the FDA. The product is now being managed by a new company, HbO_2_ Therapeutics, under a new product name (HBOC‐201) and continues to remain a subject of clinical interest.^109^ HemoLink also advanced to Phase III clinical trials but was discontinued in 2003 due to patients experiencing adverse cardiac events. In general, crosslinked and polymerized Hb based HBOCs in their clinical trials have shown risks of hypertension, microvascular dysfunction, gastro‐intestinal distress, nephrotoxicity, neurotoxicity, and increased mortality. Nonetheless, current research continues to focus on improving the manufacturability and safety profile of polymerized Hb by enhancing the molecular weight and purity of the final Hb product. For example, intramolecularly crosslinked high molecular weight polymerized Hb systems have been recently reported by academic researchers (product named PolyHb) as well as by industry entities like VirTech (product named OxyBridge).^110^, ^111^

**FIGURE 3:**
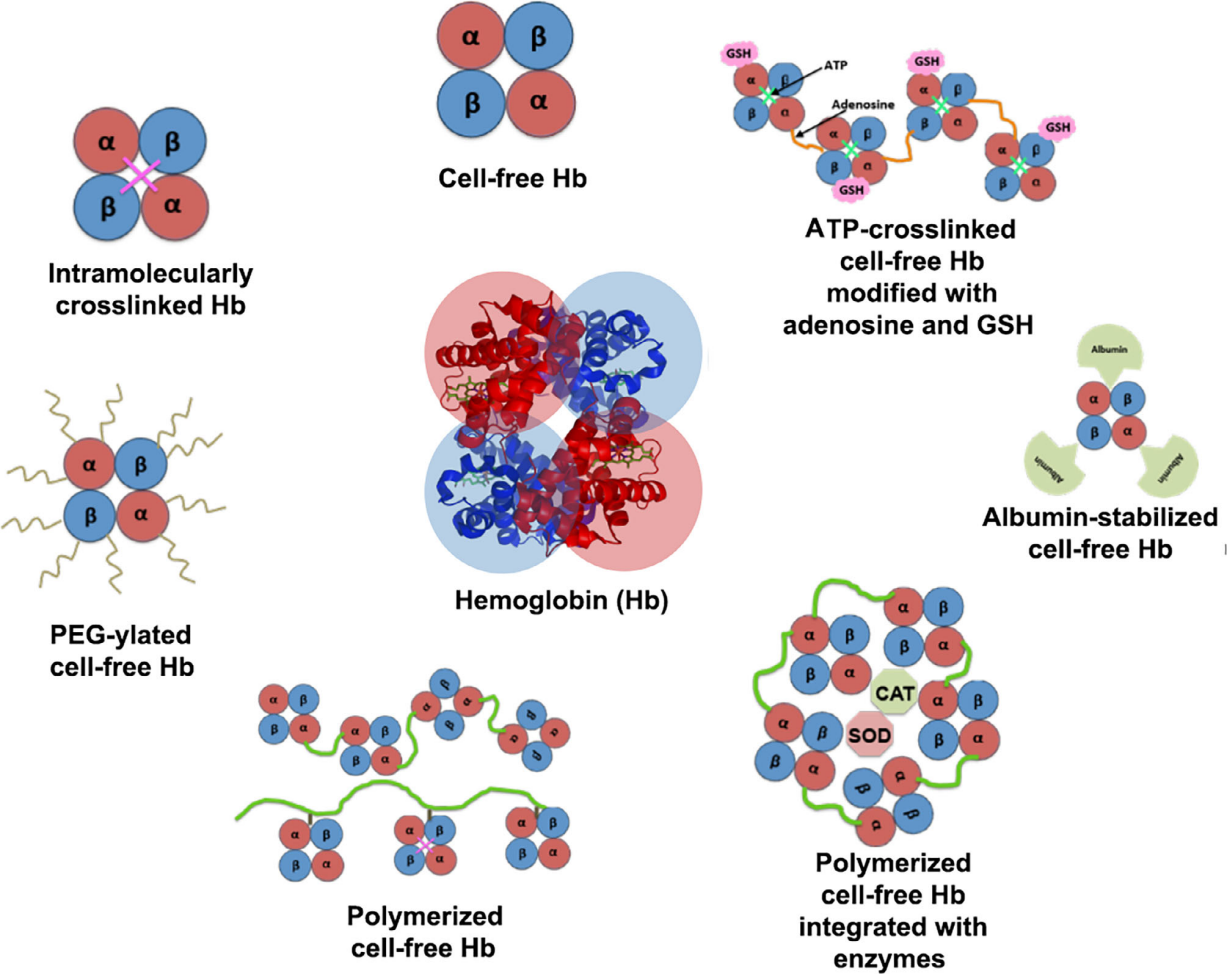
Hemoglobin (Hb) based oxygen carrier technologies utilizing cell‐free hemoglobin that is chemically modified via various biomaterials and bioconjugation approaches to improve stability, function, and in vivo safety.

Instead of crosslinking and polymerization, cell‐free Hb has also undergone modification with macromolecular bioconjugation strategies to increase circulation stability and reduce immunogenicity. In the context of macromolecular modification of biomolecules, a well‐established strategy is the bioconjugation of polyethylene glycol (PEG) to the molecule, an approach popularly known as PEG‐ylation. Since the pioneering report on PEGylation of enzymes by Frank Davis and colleagues at Rutgers University in the 1970s, this strategy has been widely applied in the modification of proteins and other biomolecules.^112^, ^113^ For HBOCs, PEG‐ylation of Hb was done for the products like Hemospan from Sangart Inc., USA and PEG‐Hb from Enzon, USA, and poly(oxyethylene) modification of pyridoxylated crosslinked Hb was done for the product PHP from Apex Bioscience, USA.^114^, ^115^, ^116^, ^117^ PEG‐ylated Hb based HBOCs have undergone extensive clinical trials and the studies demonstrated risks of bradycardia and elevation of hepatic pancreatic enzymes even at low doses. Nonetheless, the Phase I and Phase II clinical trials with Hemospan showed favorable oxygen transport in humans. However, subsequent Phase III trials in orthopedic surgery patients in Europe showed continued risks of cardiovascular and renal dysfunctions. Also, the persistent issue of NO‐scavenging by Hb continued to be a critical barrier toward the clinical success of such products.^118^, ^119^ Interesting approaches to resolve this issue include modification of Hb molecules to become NO carriers through S‐nitrosylation of cysteine residues in the *β*‐subunits of Hb or enzymatic transformation of Hb into becoming an NO donor in the presence of nitrites, but these approaches still need to be successfully evaluated for in vivo efficacy to advance toward clinical translation.^120^, ^121^ Recent research has also focused on creating reactive thiol groups away from the “heme” pocket of hemoglobin, to allow controlled site‐specific PEG‐ylation of the protein to minimize effects on the O_2_‐biding activity of the heme, but such modified Hb has not been evaluated extensively in vivo yet.^122^ Natural RBCs also contain enzymes like catalase (CAT) and superoxide dismutase (SOD) that help mitigate the oxidative stresses stemming from superoxide moieties in injured and ischemic tissues. To mimic these properties, some designs have conjugated the CAT and SOD enzymes to polymerized Hb to form PolyHb‐SOD‐CAT, which has shown combined advantages of long circulation time and reduced oxidative damage.^123^, ^124^ In yet another approach, regulatory molecules such as 2,3‐DPG and methemoglobin reductase were incorporated into cell‐free Hb based HBOC, to prevent irreversible Hb oxidation and unfavorable O_2_ affinity. In a recent design approach, purified bovine Hb was cross‐linked *intra*molecularly with ATP and *inter*molecularly with adenosine, and conjugated with reduced glutathione (GSH) to yield a product named HemoTech, with the rationale that ATP will regulate vascular tone through purinergic receptors, adenosine will counteract unwanted vasoconstriction, and GSH will protect the “heme” from reactive oxygen species.^125^, ^126^ The early phase studies have shown promising properties of HemoTech, but extensive in vitro and in vivo characterization are yet to be reported. In an interesting approach, bovine carboxyhemoglobin (CO‐Hb, instead of Hb) was PEG‐ylated and the resultant PEG‐CO‐Hb system was evaluated for oxygen (and CO) transport capabilities.^127^, ^128^ The rationale behind this approach is that endogenous CO produced from heme‐oxygenase activity can provide cytoprotective and vasoprotective effects. The PEG‐CO‐Hb product (Sanguinate, Prolong Pharmaceuticals, USA) has completed Phase I studies in healthy volunteers and has advanced to Phase II study for the reduction or prevention of delayed cerebral ischemia following subarachnoid hemorrhage. In another approach, Hb was modified by human serum albumin (HSA) by reacting Hb surface lysines to HSA cysteine‐34 using *α*‐succinimidyl‐*ε*‐maleimide cross‐linker.^129^ These core‐shell Hb‐HSA clusters are expected to have high circulation stability and can be further modified by incorporating antioxidant enzymes in the HSA pockets to protect Hb.^130^ The majority of these newer designs have only been evaluated in vitro, and rigorous preclinical in vivo studies would be needed to establish translational promise. Figure 3 shows a few of the prominent HBOC designs based on direct chemical modification of cell‐free Hb, and Figure 2c shows representative O_2_‐binding curves for such HBOC designs (e.g., Hemopure, Polyheme, and LtEc) demonstrating that these HBOCs indeed have sigmoid O_2_‐binding kinetic curves similar to native RBC, but with some differences in kinetic profiles. Table 1 provides a summary comparison of some of the prominent unencapsulated cell‐free Hb based HBOC systems in terms of materials, mechanisms, evaluation stage (preclinical and clinical) and outcomes reported, and current status as available. While such HBOC systems are yet to be FDA‐approved, ongoing research in this area highlights the continued utilization of various biomaterials‐based strategies (e.g., crosslinking, polymerization, bioconjugation, enzyme modification, etc.) to refine the structure and function of cell‐free Hb for enhancing stability, safety and efficacy.

**TABLE 1:** Comparison summary of unencapsulated cell‐free Hb based HBOCs.

Product	Material/Design	Mechanism	Preclinical evaluation	Preclinical outcome	Clinical Trial No. and Year	Primary outcome	Current status
HemAssist® (Baxter Healthcare)	Diaspirin crosslinked human Hb (DCLHb); ~64 kDa tetramer; 10–12 g/dL Hb	O_2_ transport, volume expansion	Rat, dog and swine hemorrhagic shock and ischemia–reperfusion model	Transient restoration of MAP/O_2_ delivery; impaired vaso‐activity and organ ischemia at higher doses	Phase III (NCT numbers not available)	Increased mortality, increased risk of pancreatitis and myocardial infarction in surgical patients	Development discontinued in 1999
Oxyglobin®/Hbglutamer200 (Biopure)	Glutaraldehyde‐polymerized bovine Hb; ~200–250 kDa; 13 g/dL Hb	O_2_ transport, volume expansion	Rat and dog traumatic shock model, swine hemorrhagic anemia model	Improved O_2_ content/hemodynamics, acceptable safety in canines	Approved (NCT numbers not available)	Effective for canine anemia/hemorrhage; reduced transfusion need in veterinary ER studies	Approved for dogs in US/EU. Human development not pursued.
Hemopure®/(Biopure) HBOC201 (HbO_2_ Therapeutics)	Glutaraldehyde‐ polymerized bovine Hb; ~250 kDa; 13 g/dL Hb	O_2_ transport, volume expansion	Rat, dog, and swine hemorrhagic shock model, ischemia–reperfusion, and traumatic injury model	Maintained hemodynamics & O_2_ delivery, improved survival in lethal hemorrhage	NCT01881503, 2026 NCT02684474, 2024 NCT02934282, 2024 NCT03633604, 2025	Reduced need for transfusion in anemia	Approved: South Africa (2001) & Russia (2006). US via Expanded Access only.
HemoLink®/Hemoglobin Raffimer (Hemosol)	Human Hb polymerized by oraffinose; 100–500+ kDa; 10 g/dL Hb	O_2_ transport, vasoprotection, volume expansion	Rat pharmaco‐kinetics, Rabbit hemodilution model	Improved tissue oxygenation with lower pressor response than earlier HBOCs	Phase I/II, NCT numbers not available	Reduced transfusion following coronary artery bypass; increased risk of hypertension and cardiac event	Development discontinued in 2003
PolyHeme® (Northfield Laboratories)	Human Hb polymerized with glutaraldehyde; ~150–250 kDa; 7–10 g/dL	O_2_ transport, volume expansion	Non‐human primate exchange transfusion	Improved survival; vasoactivity at higher doses	Phase III, NCT00076648 (2003–2006)	No survival benefit, increased incidence of anemia, fever, electrolyte imbalance compared to transfusion	BLA not approved; development discontinued in 2009
Hemospan®/MP4OX (Sangart, Inc.)	MaleimidePEG conjugated human Hb (PEGHb); ~90–100 kDa; 4.3 g/dL Hb	O_2_ transport, reduced NO scavenging	Rat and pig hemorrhagic shock model, non‐human primate acute anemia model	Enhanced tissue oxygenation, reduced pressor response, improved hemodynamic stability	NCT00494949, 2006 NCT00633659, 2008 NCT00421200, 2008 NCT00420277, 2008	Poor oxygenation and volume expansion	Development discontinued in 2015
OxyVita® Hb (OxyVita Inc.)	“Superpolymer” bovine Hb; ~17 MDa; 6 g/dL	O_2_ transport, reduced extravasation	Rat hemorrhagic shock model	Sustained O_2_ transport with low vasoactivity in models	Phase I completed (no public NCT)	Data not yet available	Preclinical/early clinical; ongoing research
Hemoximer®/PHP (Curacyte/Apex Bioscience)	Pyridoxylated hemoglobin polyoxyethylene (PHP), ~100 kDa; <14 g/dL Hb	O_2_ transport, reduced NO scavenging	Guinea pig, rat and swine hemorrhagic shock model	Improved O_2_ delivery, restoration of hemodynamics, favorable safety profile with minimal organ toxicity, inflammation, or oxidative damage.	Phase III NCT00021502, 2005 Phase I/II earlier NCT numbers not available	High incidence of iatrogenic distributive shock	Development discontinued in 2011
SANGUINATE® (Prolong Pharmaceuticals)	PEGylated bovine carboxy‐Hb; ~120–140 kDa; <5 g/dL Hb	O_2_ transport, reduced NO scavenging, reduced ROS production, anti‐inflammatory	Mouse stroke, ischemia–reperfusion injury model	Reduced vasoocclusion and improved perfusion	NCT01848925, 2014 NCT02411708, 2017 NCT02672540, 2017	Improved tissue oxygenation in sickle cell anemia, positive safety profile	Development paused after FDA hold
HEMO_2_life®/M101 (Hemarina, France)	Purified natural marine biopolymer, 3600 kDa; 0.1 g/dL Hb	Highcapacity O_2_ transport, ROS scavenging	Rat and pig hemorrhagic shock and ischemia–reperfusion model	Improved organ function postischemia	NCT02652520, estimated completion 2027 NCT04181710, 2023	OxyOp: safety met and less delayed graft function; OxyOp2 is safe for patients and grafts	Approved in EU as additive to preserve organs
Polymerized Hb (Palmer Lab/The Ohio State University)	Glutaraldehyde‐polymerized human/bovine Hb; ∼1000–1500 kDa; 5–15 g/dL Hb	O_2_ transport, reduced extravasation	Rat lung perfusion, guinea pig hemorrhagic shock model	Improved hemodynamics and survival	N/A	N/A	Academic preclinical development

#### 2.1.2 Encapsulation of Hb for HBOC applications

Instead of using cell‐free Hb directly in various chemically modified forms, a parallel field of research has focused on encapsulating Hb (and other associated effector and regulatory molecules) in microparticle‐ and nanoparticle‐based carrier vehicles. As mentioned previously, this approach of loading hemoglobin within a particulate carrier system was first reported in the 1950s and 1960s by Prof. Thomas Chang at McGill University, where Hb along with regulatory molecules and enzymes (e.g., 2,3‐DPG and CAT) were encapsulated within collodion (cellulose nitrate) or polyethylene glycol‐polylactide (PEG–PLA)‐based polymeric microcapsules.^66^, ^73^, ^131^ In the area of “biomaterials” research, the field of “drug delivery” has advanced tremendously over the past several decades regarding the design and use of particulate carriers that encapsulate drugs and bioactive compounds to protect them from plasma effects, improve their circulation residence time and potentially allow passive or active transport to target tissues.^132^, ^133^ The adaptation of such approaches in HBOC applications focuses on encapsulating (but not releasing) Hb within particle systems, while allowing O_2_ transport across the particle surface or shell, thereby mimicking how Hb allows O_2_ transport in and out of RBCs. Building on the work of Chang et al., subsequent designs have studied Hb encapsulation within micron and sub‐micron sized lipid vesicles (termed liposome‐encapsulated Hb or LEH), where the membrane contained phospholipids and cholesterol.^134^, ^135^, ^136^, ^137^ Several variants of this design, for example, “Neohemocytes,” “TRM‐645 Neo Red Cells,” and so forth, have been studied to maintain uniform Hb‐encapsulation, reproducible batch‐to‐batch size distribution of Hb‐loaded vesicles, optimal vesicle stability in storage and in vivo, and O_2_‐transport efficacy of the encapsulated Hb. The “Stealth Liposome” technology was established in the 1990s, where liposomal nanoparticles (100–200 nm in diameter) were surface‐functionalized with PEG to improve their size and stability while reducing their rapid macrophagic clearance in vivo.^138^, ^139^ This platform has been adapted to design Hb‐loaded PEG‐ylated liposome vesicles (HbV).^140^, ^141^, ^142^, ^143^, ^144^ In an interesting advancement of HbVs, 1,2‐dioctadecadienoyl‐sn‐glycero‐3‐phosphatidylcholine (DODPC) was used as a membrane phospholipid component, such that the sterilization of the resultant vesicles using *γ*‐irradiation produced hydroxy (OH) radicals that could then render intermolecular crosslinking of dienoyl groups to yield a highly stable liposomal membrane that could withstand severe temperature changes (e.g., lyophilization and rehydration). The HbVs reportedly contain almost double the amount of Hb compared to native RBC (~ 35 g/dL in HbV compared to ~15 g/dL in native RBC) and have shown substantial improvement of circulation lifetime (~60 h in some animal models). HbVs also reportedly reduce the NO‐scavenging issues and renal toxicity issues that are otherwise associated with cell‐free Hb designs. This design continues to be rigorously evaluated as an intravenously administered “RBC surrogate” in several preclinical hemorrhage models in vivo.^145^, ^146^, ^147^, ^148^ Recently, an interesting report also described the administration of HbVs via the intraosseous route in rabbits to provide RBC‐mimetic hemodynamic distribution into circulation.^149^ Analogous designs using encapsulation of Hb into liposomes of a different composition than HbV have also been reported, for example, liposome‐encapsulated hemoglobin (LEH) system.^150^, ^151^ Successful translational advancement of HbV and LEH systems via pre‐clinical and clinical studies could provide an effective biosynthetic RBC surrogate for transfusion applications in perioperative settings, anemia, hemodilution, acute traumatic coagulopathies with massive hemorrhagic shock, oxygenation of ischemic tissues, and ex vivo preservation of organ transplants.

Besides encapsulation in liposomal platforms, Hb has also been loaded in other biomaterials‐based particle systems, especially made of polymers like PEG–PLA, poly(ε‐caprolactone)/poly (L‐lactic acid) (PCL/PLA), poly (lactic‐co‐glycolic acid) (PLGA)/PEG copolymers, and so forth.^152^, ^153^, ^154^ Amphiphilic block‐copolymer systems form liposome‐analogous polymeric vesicles, also known as polymersomes, that are widely studied in the *drug delivery* research area since their seminal report by Dennis Discher, Daniel Hammer, and colleagues in 1999.^155^, ^156^, ^157^ Hb has been incorporated in such polymeric vesicle systems leading to polymersome‐encapsulated Hb (PEH) products.^158^, ^159^, ^160^, ^161^ Reportedly, Hb loading in these PEH systems is usually at 1–2 mg/mL, which is much lower compared to RBCs. These PEH systems can allow O_2_‐transport similar to RBCs, but extensive in vivo evaluation of these systems is currently lacking. In a recent approach utilizing polymer particles, a multi‐layer assembly construct was made starting with a PLGA core, then decorating the core with poly(dopamine)‐coated Hb, then assembling cerium oxide nanoparticles around the Hb layer to enable reactive oxygen species (ROS) depletion, and finally decorating the assembly surface with a PEG brush to render steric stability and prevent non‐specific protein adsorption.^162^ The poly(dopamine)‐coated Hb in these constructs demonstrated O_2_‐binding kinetics similar to natural Hb, and the constructs themselves showed favorable biocompatibility, hemocompatibility, and antioxidant properties. However, the scalability of these complex multilayer constructs as well as their in vivo efficacy to render tissue oxygenation in shock and hemorrhagic settings are yet to be reported. Polymer capsule systems encapsulating Hb have also been reported using hydrogel‐based capsules, but as with PEH systems, these designs currently remain in the in vitro evaluation stages only, to optimize their morphological and bioactive functions.^163^, ^164^, ^165^ Hb‐loaded particle systems have also been created by co‐precipitating Hb with calcium carbonate (CaCO_3_), followed by crosslinking with glutaraldehyde and selective dissolution of CaCO_3_, to form Hb‐encapsulated microparticles.^166^ Similar Hb‐loaded microparticles carrying about 80% Hb content compared to natural RBCs have also been reported, where Hb and manganese carbonate (MnCO_3_) were co‐precipitated, followed by HSA‐mediated stabilization.^167^ These particles have shown reduced NO scavenging effects on vasoconstriction. Some approaches have also directly conjugated Hb to the hydrophobic or hydrophilic domain of block‐copolymers, to then form Hb‐loaded micelles via self‐assembly.^168^, ^169^ In another approach, MnCO_3_ nanoparticles were used as templates for layer‐by‐layer (L‐B‐L) deposition of Hb and dialdehyde heparin (DHP), followed by layer stabilization with crosslinking and then selective dissolution of the MnCO_3_ core to form Hb‐loaded particles.^170^ A similar approach was also used with layers of Hb, DHP, and the enzyme catalase (CAT), to create L‐B‐L assembled Hb‐loaded nanotubes that reportedly exhibit the redox properties of CAT.^171^ These newer Hb‐encapsulated polymeric and inorganic particle designs have been characterized in vitro for their morphology, stability, cytotoxicity, and O_2_‐binding capacity, but they remain suboptimal in terms of RBC‐mimetic Hb loading and O_2_ transport capabilities, and in vivo evaluation of these systems for safety and efficacy is currently significantly lacking. In a more recent approach, a phospholipid‐polymer‐peptide amphiphile‐based hybrid vesicular nanoparticle structure encapsulating Hb, named Erythromer, has been reported.^172^, ^173^ Reportedly, this design has a tunable membrane that enables pH‐responsive electrostatic interaction with small allosteric effector molecules to render O_2_‐affinity modulation. The design also incorporates redox molecules such as leucomethylene blue in its “shell” to inhibit methemoglobin (metHb) formation and thus protect the encapsulated Hb. Additionally, the design incorporates low molecular weight PEG and the carbohydrate‐based lyoprotectants, such that Erythromer can be potentially lyophilized and stored as a powder, to be aqueous reconstituted on‐demand. Preliminary safety, efficacy, and pharmacokinetic studies of this unique multi‐component RBC surrogate have been recently reported in rat and rabbit models, and continued evaluation in appropriate animal models of RBC dysfunctions and traumatic coagulopathies is expected to advance toward translation. In another interesting approach, some studies have attempted to encapsulate O_2_ directly (instead of Hb) within phospholipid microvesicles (2–4 μ in diameter), to enable O_2_‐delivery to deoxygenated RBCs in circulation.^174^, ^175^ These O_2_‐loaded microbubbles were found to be stable for a few weeks in storage with minimal oxygen loss; however, in vivo, they showed a very short circulation lifetime (<1 h), which may be a critical barrier to clinical translation.

**FIGURE 4:**
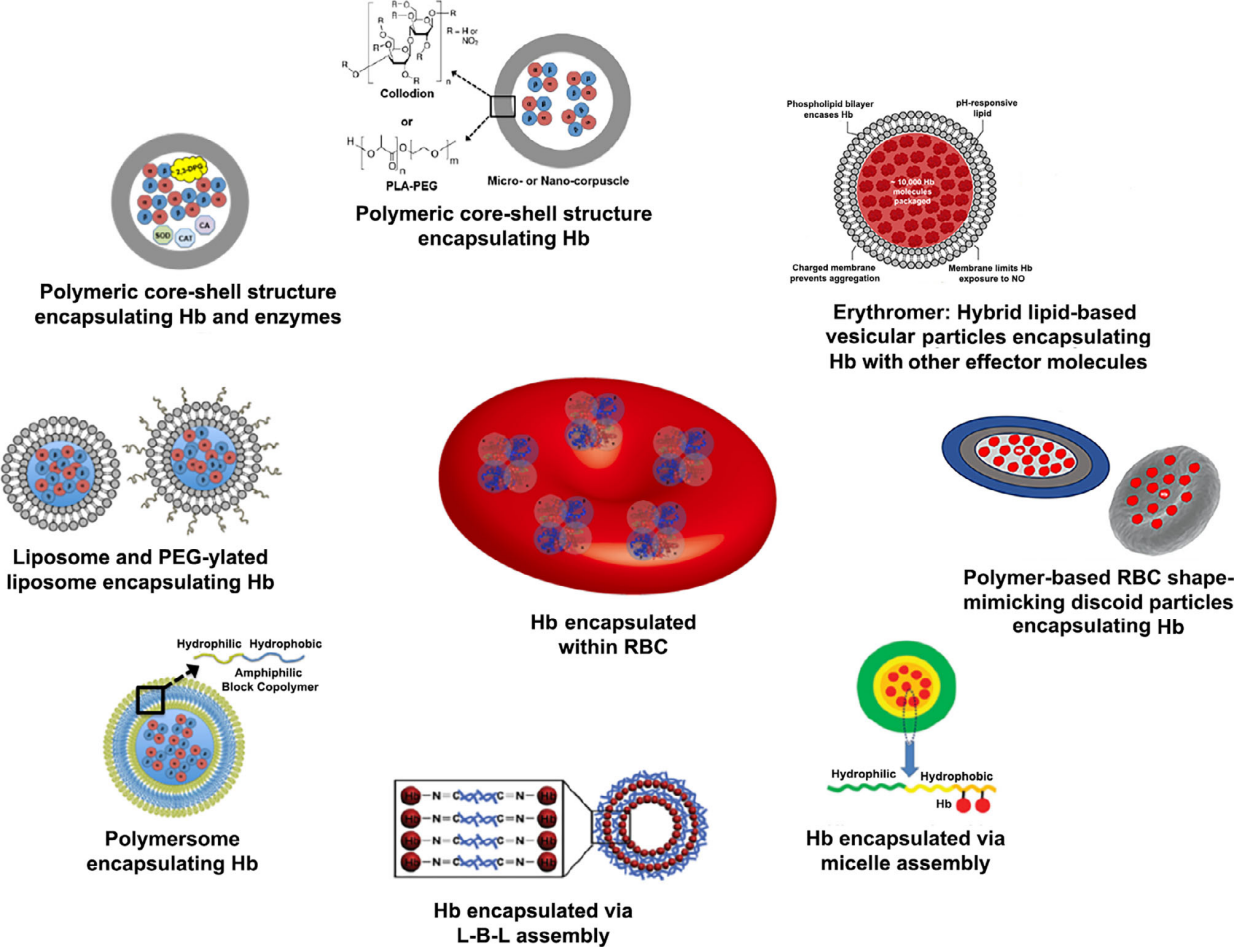
Hemoglobin (Hb) based oxygen carrier technologies utilizing encapsulated hemoglobin where various biomaterials‐based carrier platforms are loaded with hemoglobin and other regulatory molecules to improve hemoglobin stability, function, and in vivo safety.

An emerging and exciting area of *drug delivery* research is the design of biomaterials‐based particulate vehicles that mimic the biophysical properties of cells, namely, morphology and biomechanical properties, that influence their functions.^176^, ^177^, ^178^, ^179^, ^180^ In this context, morphological and biomechanical properties of natural RBCs are known to significantly influence their margination in hemodynamic flow, their interactions in tissue capillaries, and their oxygen transport functions.^181^, ^182^ Healthy RBCs are biconcave discoid, ~8 μm in diameter and ~2 μm thickness, and are highly flexible (Young's Modulus 0.1–0.2 kPa), which enables their efficient passage through microvascular circulation for oxygen transport. Based on these natural cues, RBC‐inspired size, shape, and flexibility parameters have been mimicked in Hb‐encapsulating particles. For example, a polyelectrolyte driven L‐B‐L assembly approach has been used to create particles that mimic the shape and deformability of natural RBCs. In one such approach, Hb and BSA were electrostatically deposited on the surface of RBC‐shaped PLGA particles of ~7 μm diameter and 400 nm shell thickness, and then the PLGA core template was dissolved to yield RBC shape‐mimicking Hb‐loaded flexible microparticles.^183^ Similar RBC‐inspired particles have been designed utilizing a stop‐flow‐lithography (SFL) approach with PEG hydrogels pioneered by the Doyle laboratory, where the mechanical properties of the particles were controlled by modulating the cross‐linking density of the hydrogel.^184^ In another exciting approach pioneered by the DeSimone laboratory, RBC‐inspired polymeric microparticles were manufactured from acrylate hydrogels using a “particle replication in nonwetting templates” (PRINT®) technology and loaded with Hb.^185^ These Hb‐loaded particles demonstrated RBC‐mimetic elastic deformation in vitro in microfluidic systems. In yet another approach, liposome‐encapsulated actin‐hemoglobin (LEAcHb) particles, ~140 nm in diameter, were manufactured using a polymerized actin core, and these particles exhibited RBC‐mimetic shape and flexibility, which improved their circulation half‐life.^186^ In healthy RBCs, the negative surface charge prevents RBC aggregation over a distance of 20 nm, and this aspect has led to the mimicry of RBC‐relevant surface charge on Hb‐encapsulating PEG–PLA nanoparticles (<200 nm in diameter) using cetyltrimethylammonium bromide (CTAB) or anionic sodium dodecyl sulfate (SDS) as surfactants.^187^ Interestingly, cationized particles were found to have a half‐life of ~11 h (8‐fold higher than untreated particles), while the anionized particles were quickly eliminated, giving a half‐life of <1 h. In recent years, a unique area of biomaterials‐based drug delivery research has focused on the approach of *biointerfacing*, which involves the coating of synthetic microparticles and nanoparticles with extracted cell membrane to impart biointeractive properties.^188^, ^189^, ^190^ In this area, recent research has reported on utilizing natural RBCs as a template to build “synthetic RBC” particles via silicification, polymer deposition, and RBC‐derived membrane coating, and loading with Hb.^191^ In another “biointerfacing” approach, Hb was coated with poly‐lysine, complexed with cerium oxide‐based antioxidant particles, and these complex constructs were coated with extracted RBC membrane.^192^ The resultant membrane‐interfaced constructs showed O_2_‐binding and release capabilities, as well as biocompatibility and antioxidant properties in vitro. The various above‐described approaches to mimic RBC geometry, morphology, and flexibility in Hb‐encapsulated particulate RBC surrogate development have all shown promising in vitro properties, but their ability to load RBC‐equivalent Hb concentrations and their in vivo safety and efficacy at therapeutic doses regarding O_2_‐transport for transfusion applications are yet to be evaluated rigorously. Figure 4 shows important representative designs and approaches for HBOCs developed via encapsulation of Hb in biomaterials‐based particle constructs to render “RBC surrogate” systems, and Table 2 shows a summary comparison of some of these designs in terms of evaluation and current status.

**TABLE 2:** Comparison summary of selected HBOCs utilizing encapsulation of Hb in particulate platforms.

Product	Material/Design	Mechanism	Preclinical evaluation	Preclinical outcome	Clinical trial No. and year	Primary outcome	Current status
Hemoglobin vesicles (HbV)	Human Hb, PEGylated phospholipid shell, 10–35 g/dL Hb	O_2_ diffusion through lipid vesicle membrane	Rat hemodilution and blood exchange model; Rat, rabbit and dog hemorrhagic shock model; Rat uncontrolled hemorrhage	Improved hemodynamics, resuscitation and survival (where applicable) compared to control	jRCT2011200004, 2022	Generally safe for human administration up to 50 mL; inflammation and liposome‐induced infusion reactions (fever and elevated C‐reactive protein) at high doses	Efficacy trial reported to begin in 2025
Erythromer (Kalocyte)	Human Hb with various effector molecules and lyoprotectant, PEGylated lipid shell, 5–10 g/dL Hb	Effector‐assisted pH‐responsive membrane diffusion exchange of O_2_	Rat hemorrhagic shock model, pharmaco‐kinetics in rats and rabbits	Improved tissue pO_2_ and survival and reduced NO scavenging compared to RBCs; clearance in 8–10 h	Pending NCT05756426, estimated completion 2029	Pending	Pre‐clinical
Hemoglobin corpuscles	Bovine Hb with effector molecules, nitro‐cellulose or PEG/PLA shell, ~5 g/dL Hb	Effector‐assisted membrane diffusion of O_2_	Mouse hemorrhagic shock and ischemia–reperfusion model	Poor circulation time, acute toxicity after multiple doses, reduced tissue damage after ischemia	N/A	N/A	Not under studies currently
Liposome‐encapsulated hemoglobin (LEH), neohemocytes, TRM‐645 Neo Red Cells	Bovine or human Hb, PEGylated phospholipid shell, 6 g/dL Hb	Effector‐assisted membrane diffusion of O_2_	Mouse safety injections, rat hemorrhagic shock model	Reduced complement activation, high circulation time, no toxicity after multiple doses, improved organ health after shock	N/A	N/A	Pre‐clinical
Polymer‐encapsulated hemoglobin (PEH)	Bovine Hb with effector molecules, polymeric shell (PLL, PCL/PLA, or PLGA/PEG) 10–15 g/dL Hb	Membrane diffusion of O_2_ controlled by polymer shell thickness	Mouse safety injections and pharmaco‐kinetics	Improved circulation time, no organ/tissue damage	N/A	N/A	Pre‐clinical

### 2.2 Perfluorocarbon (PFC)‐based oxygen carrier systems

In parallel to the research and development efforts in HBOCs made with chemically modified cell‐free Hb and encapsulated Hb systems, studies have also focused on the development of “fully synthetic” oxygen carrier systems that do not use Hb as the O_2_‐binding molecule. The major class of compounds used for these approaches is perfluorocarbons (PFCs). PFCs are biologically inert, and liquid PFCs are well‐established in the clinic as contrast agents for ultrasound imaging.^193^, ^194^, ^195^ Interestingly, O_2_ can loosely bind to PFCs via van der Waals type interactions, with the binding kinetics following Henry's law, and this results in a linear O_2_‐binding profile compared to the positively allosteric sigmoid O_2_‐binding curve of Hb.^196^, ^197^, ^198^ Such a linear O_2_‐binding curve for PFCs implies that a much higher O_2_ partial pressure (pO_2_) is required to saturate PFCs with oxygen, which is a mechanistic disadvantage compared to Hb. However, due to the linear profile, oxygen de‐saturation (i.e., O_2_ release) ability is better for PFCs compared to Hb. Since PFCs are immiscible in water, they need to be emulsified with suitable surfactants (e.g., phospholipids) for intravenous in vivo applications while ensuring that the surfactant concentration does not affect the O_2_‐binding property.^199^ Several PFC‐based emulsions have undergone rigorous investigation as oxygen carriers. For example, Fluosol‐DA (Green Cross Corp, Japan), an emulsion of 10%–20% perfluorodecalin in albumin, was tested in several animal models and progressed to clinical trials to receive FDA approval in 1989.^200^

**FIGURE 5:**
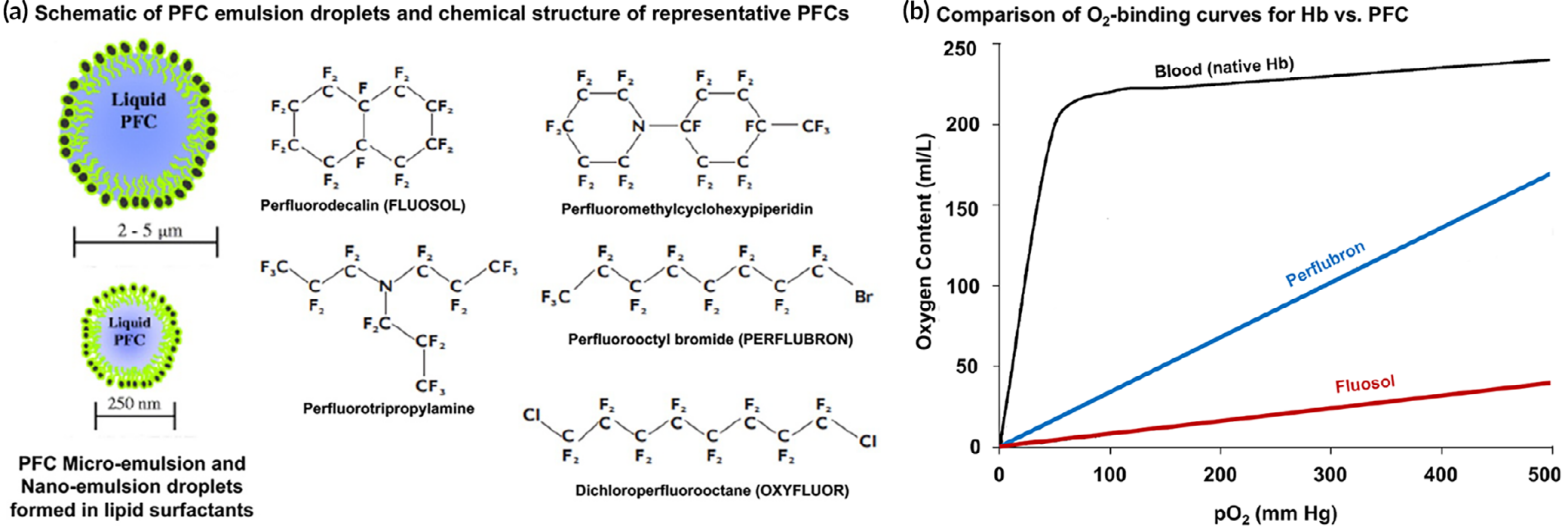
(a) Representative chemical structures of perfluorocarbon (PFC) molecules with oxygen‐binding capability, which can form microemulsion or nanoemulsion droplets stabilized by lipidic surfactants; (b) Characteristic oxygen binding profile of hemoglobin (sigmoid) compared to that of PFC (linear) indicating that compared to oxygen partial pressure (pO_2_) required to saturate hemoglobin, a much higher pO_2_ is required to saturate PFCs.

However, this product was soon recalled because of issues like low O_2_ transport capacity (0.4 mL O_2_/100 mL compared to O_2_ transport capacity of 20.1 mL/100 mL in natural blood), premature oxygen release, short shelf life, temperature instability, slow excretion, and negative biological side effects like low platelet counts (thrombocytopenia), febrile symptoms, and macrophage activation (inflammatory trigger). Newer generation PFCs like perfluorooctyl bromide (Perflubron), perfluorodecyl bromide, and perfluorodichlorooctane reduced some of these issues associated with Fluosol.^201^, ^202^, ^203^, ^204^, ^205^, ^206^, ^207^ Specific ratios of these compounds were combined with egg‐yolk phospholipids to develop an oxygen carrier emulsion named Oxygent (Alliance Pharmaceutical Corp, USA), that demonstrated reduced macrophage activation.^202^, ^203^ Oxygent droplets showed a circulation half‐life of ~10 h at a dose of 1.8 g/kg in humans.^208^, ^209^, ^210^ Oxygent could also render higher O_2_‐delivery compared to Fluosol, but this was still ~30% less efficient compared to natural RBCs. A similar PFC emulsion product was developed using perfluorodichlorooctane, egg yolk phospholipid, and triglyceride, named Oxyfluor (HemaGen, USA), and this showed properties similar to Oxygent.^204^ The ability of nanoscale PFC droplets to occupy small plasma volumes and circulate efficiently through microcapillaries for O_2_‐transport was considered an advantage. In preclinical evaluation using appropriate animal models, both Oxygent and Oxyfluor showed promising outcomes in O_2_‐delivery, but both products also showed thrombocytopenic side‐effects.^209^, ^210^ In several large animal (e.g., canine) models of hemodilution and cardiopulmonary bypass, these PFC‐based oxygen carriers showed promising ability to improve tissue oxygenation, and in fatal hemorrhagic shock models, these PFC products improved resuscitation outcomes and reduced mortality. These preclinical results have led to the clinical evaluation of these PFC products in humans undergoing major non‐cardiac surgery, hemodilution, and hemorrhage.^211^, ^212^, ^213^ Oxygent was able to render effective O_2_‐transport and reduce the need for autologous blood transfusion, indicating their promise as an RBC surrogate technology. However, further clinical trials of such products in cardiopulmonary bypass patients were terminated in the United States because of increased risk of adverse cardiovascular events and stroke, although closer analysis of the data suggests that these adverse events may have been associated more with erroneous study protocol rather than the PFC product itself. Oxycyte (Oxygen Biotherapeutics Inc., USA), a third generation PFC emulsion containing perfluoro‐tert‐butylcyclohexane and egg yolk phospholipids, has also undergone evaluation in several animal models and then clinical trials in patients with traumatic brain and spinal cord injury.^214^, ^215^, ^216^ Although some safety concerns were raised regarding Oxycyte's effect on the immune system, recent animal studies have alleviated these concerns. Perftoran (Perftoran, Russia) is another PFC emulsion made with perfluorodecalin and perfluoromethylcyclopiperidine in a non‐ionic surfactant that has been widely investigated and used in Russia.^217^, ^218^ This product was reported to show adverse effects like hypotension and pulmonary complications, but randomized clinical trials with Perftoran conducted in Mexico City on patients undergoing cardiac valvuloplasty showed higher intraoperative pO_2_ levels and reduced need for allogenic RBC transfusion. In recent years, the approach of biointerfacing (coating of particles with cell membrane) has also been explored for PFC emulsions, especially via utilization of RBC‐derived membranes to encapsulate PFC droplets.^219^, ^220^ While such biosynthetic PFC emulsion designs have shown oxygen transport potential in mouse models in these studies, their resuscitative capacity in hemorrhagic shock and major traumatic coagulopathy settings, as well as their reproducible manufacturing scalability for efficient translational advancement, are yet to be studied. Figure 5a shows chemical structures of some important PFC‐based O_2_‐carrier products and Figure 5b shows the comparison of O_2−_binding kinetic profiles of representative PFC products versus native Hb.

### 2.3 Synthetic porphyrin‐based oxygen carrier systems

**FIGURE 6:**
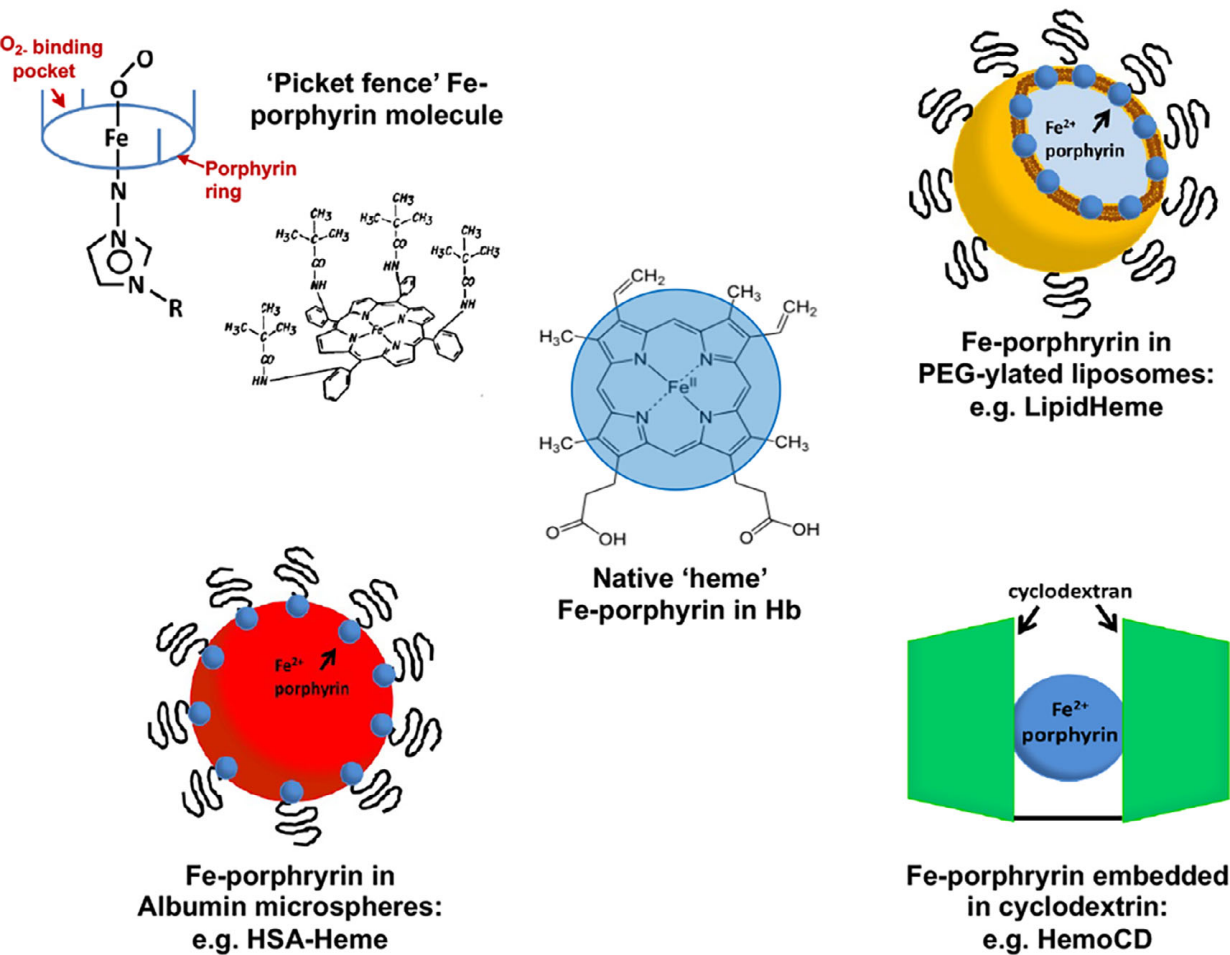
Various biomaterials‐based designs incorporating iron porphyrin systems for oxygen carrying applications.

From a chemical structure standpoint, the O_2_‐binding “heme” pocket of Hb in RBCs is made of a porphyrin tetrapyrrole ring surrounding an iron atom (Figure 2a). Based on this, several research approaches have focused on evaluating Fe (II)‐bearing porphyrin systems directly for oxygen transport.^221^ For example, the development of “picket fence” Fe^2+^ porphyrin molecules demonstrated reversible oxygenation of myoglobin and hemoglobin via immobilization of ferrous “heme” in a sterically hindered hydrophobic matrix. While these systems showed cooperative O_2_‐binding similar to Hb, they were prone to irreversible oxidation in an aqueous environment. To address this, a hydrophobic environment was created to protect these molecules by encapsulation in liposomes, where amphiphilic Fe^2+^‐porphyrin bearing four alkylphosphocholine groups were embedded into the phospholipid bilayer, resulting in a product named LipidHeme.^222^, ^223^, ^224^ These vesicles reportedly demonstrated reversible binding and release of oxygen similar to Hb, but further translation of this technology has not been reported. In an analogous design, Fe (II)‐containing porphyrin systems were incorporated in HSA particles, and these have shown RBC‐mimetic O_2_‐transport capability. Surface PEG‐ylation of these particles has enabled increased circulation time. In another approach, a 1:1 complex of 5,10,15,20‐tetrakis (4‐sulfonatophenyl) porphinatoiron (II) (Fe (II) TPPS) and a per‐*O*‐methylated β‐cyclodextrin dimer having a pyridine linker (termed HemoCD or Py3CD) was prepared.^225^, ^226^ This system has shown oxygen affinities similar to natural Hb but was found to undergo gradual autoxidation in an aqueous environment. The circulation stability of these systems could be further enhanced by surface decoration with PEG motifs. Figure 6 shows representative Fe‐porphyrin based designs that are under research for oxygen transport applications. These various porphyrin‐based oxygen carrier designs form exciting biomaterial approaches as RBC surrogates for O_2_ transport, but currently, they lag behind HBOCs and PFC‐based O_2_‐carrier systems in translational advancement.^227^

## 3 PLATELET SURROGATES AND HEMOSTATIC BIOMATERIALS

**FIGURE 7:**
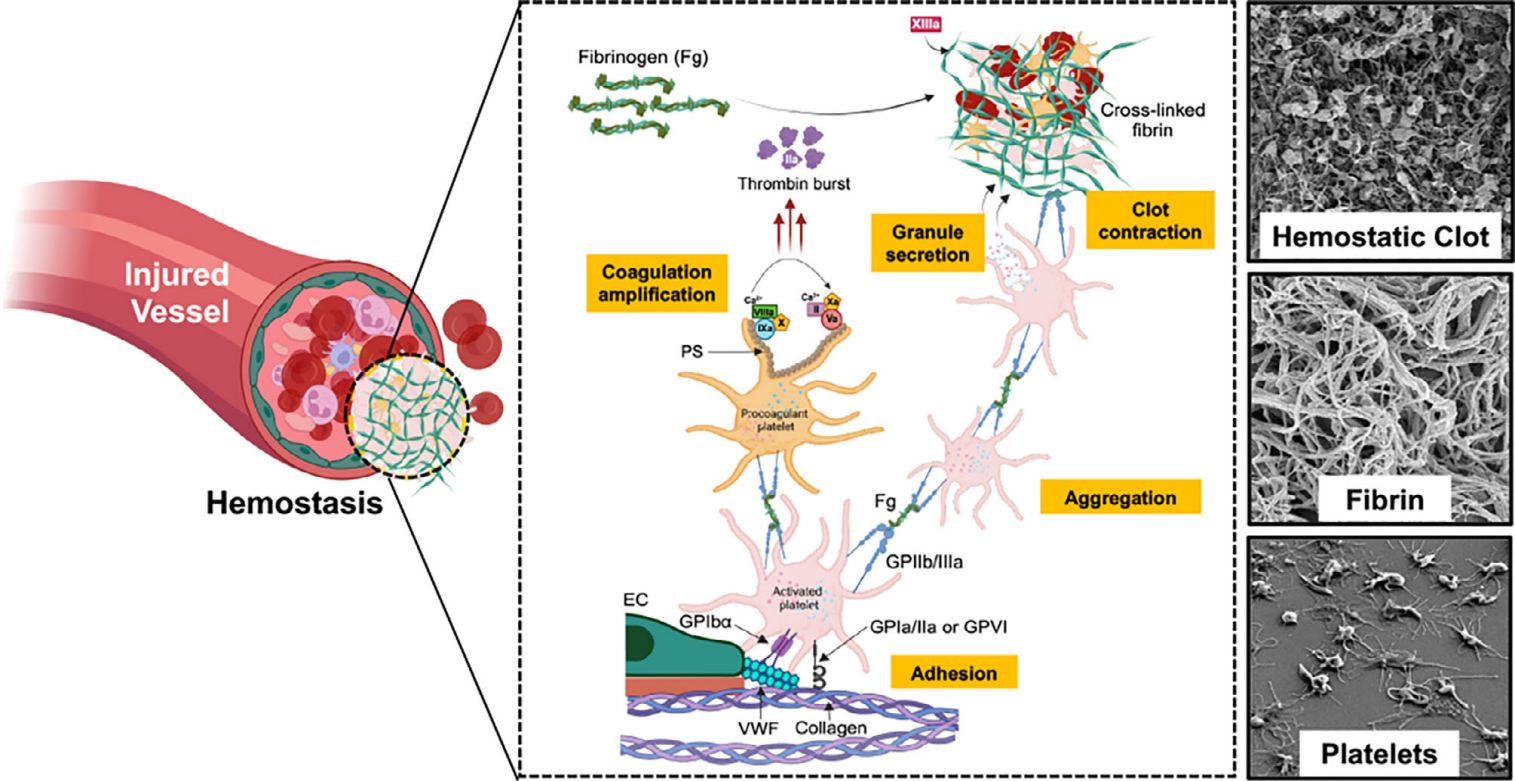
Schematic of hemostasis mechanisms involving platelet adhesion and aggregation, platelet‐mediated coagulation amplification for thrombin production and fibrin generation, and platelet granule secretion for augmenting coagulation outcomes and fibrin stability; representative scanning electron microscopy (SEM) images of activated platelets, fibrin, and final hemostatic clot are shown.

Platelets are anucleate cells produced from mature megakaryocytes in the bone marrow and released in blood circulation. In humans, a healthy platelet count is 150,000–400,000 per μL and their circulation lifespan is 7–9 days. Hemostasis is the body's natural process of *necessary* blood clotting at the site of a vascular injury to prevent blood loss from the body.^228^, ^229^ Platelets play a multifaceted role in this complex process. The hemostatic response of platelets at a bleeding injury site occurs via multiple concomitant mechanisms (Figure 7).^230^, ^231^, ^232^, ^233^, ^234^, ^235^, ^236^, ^237^, ^238^, ^239^, ^240^, ^241^, ^242^, ^243^, ^244^, ^245^, ^246^, ^247^, ^248^, ^249^, ^250^ These include: (1) Rapid *adhesion* to von Willebrand Factor (VWF) and collagen exposed at the injury site to initiate “platelet plug” formation; (2) *Activation* and fibrinogen (Fg)‐mediated *aggregation* of activated platelets at the site to grow the platelet plug; (3) Presentation of anionic phospholipids like phosphatidylserine (PS) on the surface of procoagulant activated platelets to allow coagulation factor co‐localization for *thrombin amplification*; (4) *Secretion* of several clot‐promoting and clot‐stabilizing molecules from cytoplasmic granules (e.g., vWF, Adenosine diphosphate or ADP, inorganic polyphosphate or PolyP, etc.) and membrane lipid processes (e.g., thromboxane A2 or TXA_2_) to enhance the kinetics and stability of blood clotting; and (5) Facilitating *clot retraction* via contractile forces induced by the binding of platelet surface integrin GPIIb‐IIIa to fibrin. Therefore, depletion of circulating platelet count caused by reduced production or increased consumption, as well as loss of platelet function due to disease‐induced, drug‐induced, or congenital defects, results in bleeding complications and coagulopathies. Consequently, platelet transfusions are used routinely in the clinic to prevent bleeding risks (prophylactic transfusion) and reduce active hemorrhage (emergency transfusion), with approximately 7000units of platelets transfused annually in the United States.^251^, ^252^, ^253^ These transfusions use allogeneic platelets collected by either pooling from multiple donors or apheresis from a single donor, and these donor platelet suspensions are currently stored at room temperature (20–24°C) with gentle agitation, as per FDA guidelines (FDA‐2014‐D‐1814). However, in such storage conditions, platelets have a high risk of bacterial contamination, which results in their very short shelf‐life (~5–7 days).^253^, ^254^, ^255^, ^256^ Additionally, “donor shortage” remains a persistent issue, more so evident during challenging times like the recent COVID‐19 pandemic when blood donations have been scarce.^23^, ^257^ The combination of “insufficient availability” and “short shelf‐life” poses severe challenges regarding timely utilization of platelet transfusions within hospitals, as well as in pre‐hospital settings at point‐of‐injury (POI) and *en route* bleeding management in civilian and battlefield scenarios. Significant endeavors are currently ongoing to improve the storage‐life and availability of platelets, via utilization of unique PRTs, as well as use of reduced temperature processing and storage, for example, chilling platelets to 4°C (cold‐stored platelets or CSP), cryopreserving platelets, freeze‐drying of platelets (e.g., the product Thrombosomes by CellPhire, USA), and so forth.^258^, ^259^, ^260^, ^261^, ^262^, ^263^, ^264^, ^265^, ^266^, ^267^, ^268^, ^269^, ^270^, ^271^, ^272^, ^273^, ^274^ While these approaches are expected to improve platelet transfusion logistics, they are still dependent upon “donor platelet” availability, which continues to be a persistent challenge. To address this issue, two parallel efforts have emerged. One approach involves the utilization of unique growth factors in innovative bioreactor scaffold systems to create *biologic* platelets in vitro from stem cells, as was discussed earlier in this article. The complementary and parallel approach is the utilization of bioinspired engineering to create *synthetic* platelet surrogates that can mimic platelets' mechanisms of hemostasis while allowing large‐scale manufacture, effective sterilization, and longer shelf‐life, and here biomaterials are playing a central part.^275^, ^276^, ^277^

### 3.1 Design of synthetic platelet surrogates using biomaterials‐based particulate systems

Analogous to the approaches described previously in designing functional mimics of RBCs utilizing lipidic and polymeric particulate systems, a significant amount of research has been directed at designing particle‐based platelet‐mimetic synthetic hemostatic technologies. The first response of natural platelets to a bleeding injury site is their rapid adhesion under hemodynamic flow to exposed VWF and collagen at the site.^233^, ^234^ VWF is secreted from injured endothelium as a globular protein and under hemodynamic shear it unravels to expose several biointeractive domains.^278^ Of these, the A1 domain of unraveled VWF allows binding of platelets via platelet surface receptor component GPIbα of the GPIb‐IX‐V complex. The A3 domain of unraveled VWF binds to subendothelial collagen and thus the VWF molecule forms a bridging surface, much like a double‐sided tape, between platelets and collagen. Platelets can also directly bind to the exposed collagen via their surface glycoproteins GPIa‐IIa and GPVI. Thus, the synergistic mechanisms of platelet adhesion to VWF and collagen allow for a large number of platelets to rapidly localize at the injury site, initiating the formation of the platelet plug. These adhesion mechanisms have inspired biomaterials‐based design of “synthetic platelet” constructs that emulate platelets' VWF‐binding and collagen‐binding capabilities. An early design called “Plateletosomes” involved detergent‐based extraction of platelet membrane glycoproteins and their subsequent incorporation within the lipid membrane of liposomes.^279^ A variation of this approach resulted in the technology called *infusible platelet membrane* (IPM, Cypress Bioscience), that utilized extraction of natural platelet membrane, pasteurization and formation of lyophilized vesicles therefrom.^280^, ^281^ While both products showed promising hemostatic properties, they did not achieve clinical translation. As discussed previously, several recent research studies have reported the utilization of extracted platelet membranes to coat synthetic polymer nanoparticles (*biointerfacing*), demonstrating that some of the surface glycoprotein functionalities may be conserved on these extracted membranes, to allow platelet‐relevant biointeractions of the coated nanoparticles.^282^, ^283^, ^284^ While these approaches are scientifically elegant, the extraction, purification, particle incorporation/interfacing steps and immune compliance aspects required for such approaches can make such strategies potentially too complex to scale up while preserving batch‐to‐batch quality and functional fidelity. This can become a critical barrier to clinical translation while navigating a complicated regulatory pathway due to the presence of both bioderived and synthetic components in the product. Additionally, such approaches necessitate availability of donor‐derived platelets (for membrane extraction) and thus may suffer from the already existent challenges of the limited availability and shelf‐life of donor platelets that were discussed in the previous section.

Instead of utilizing platelet‐derived membrane components, evolved versions of this approach have utilized the bioconjugation of recombinant GPIbα (rGPIb*α* that binds to VWF's A1 domain) and GPIa‐IIa (rGPIa‐IIa, that binds to collagen) on the surface of liposomes, latex beads, or albumin‐based particles.^285^, ^286^, ^287^ In further advancement of this approach, both rGPIb*α* and rGPIa‐IIa were co‐conjugated on the surface of liposomes and albumin particles, and this combination demonstrated higher binding to collagen surfaces in the presence of soluble vWF in high shear flow environments, closely mimicking the adhesion capabilities of natural platelets.^288^ One potential challenge for this approach is the large size of the recombinant protein fragments, which, upon co‐decoration on the surface of nano‐ or microparticles, can pose mutual steric interference and particle destabilization. This issue can be potentially resolved by using small peptides that have vWF‐binding and collagen‐binding capabilities. An early example of this is found in reports where a GPIb*α*‐relevant 15‐mer peptide was decorated in multiple copies on a liposomal platform to enable binding ability to vWF.^289^ Although the biochemical properties of these constructs were characterized, the evaluation of the actual hemostatic efficacy of this system has not been reported. In our research in this area, we have utilized a vWF‐binding peptide (VBP) sequence TRYLRIHPQSWVHQI derived from the C2 domain (residues 2303–2332) of the coagulation factor FVIII and a collagen‐binding peptide (CBP), which is a 7‐mer repeat of the Glycine (G)‐Proline (P)‐Hydroxyproline (O) tri‐peptide (i.e., –[GPO]_7_–) with helicogenic affinity to fibrillar collagen but minimal affinity to platelet collagen receptors GPIa/IIa and GPVI, to mimic platelet adhesion mechanisms on liposomal and albumin‐based nanoparticles.^290^, ^291^ Liposomes co‐decorated with VBP and CBP motifs showed significant platelet‐mimetic adhesion on “VWF + collagen” surfaces in microfluidic channels at low‐to‐high shear ranges, mimicking hemostatically relevant platelet adhesion. This approach emphasizes the utilization of peptide combinations for *heteromultivalent* functionalization of nanoparticles for platelet‐mimetic synergistic bioactivity. In fact, the design benefit of such *heteromultivalent* decorations in augmenting the biofunctional output of synthetic particles has also been demonstrated in other areas of biomaterials research, for example, mechanistic studies of leukocyte rolling and adhesion conducted by Hammer and Eniola‐Adefeso laboratories utilizing polystyrene particles co‐decorated with selectin‐binding and ICAM‐1‐binding ligands, targeting of nanoparticles to activated endothelium via combination binding of E‐selectin and VCAM‐1 conducted by Auguste laboratory, and research conducted in our laboratory on combination targeting of multiple receptors on platelets and “platelets + fibrin” for enhancing nanoparticle targeting in vascular drug delivery.^292^, ^293^, ^294^, ^295^, ^296^, ^297^

While rapid adhesion to exposed VWF and collagen is the earliest hemostatic response of natural platelets, in order to form a stable hemostatic plug, the site‐activated platelets undergo aggregation via fibrinogen (Fg)‐mediated bridging of the stimulated conformation of integrin GPIIb‐IIIa on platelet surface.^298^, ^299^, ^300^ Fg binds to integrin GPIIb‐IIIa on activated platelets via interaction with the RGD peptide sequences present in the *α* chain and the HHLGGAKQAGDV peptide sequence (also known as H‐12 peptide) present in the *γ* chain of Fg at both termini, thus essentially *crosslinking* platelets. Based on this mechanism, several “synthetic platelet” approaches have aimed to mimic this by coating synthetic particle surfaces with Fg itself, Fg fragments, or Fg‐based RGD and H‐12 peptides. Earliest examples of this approach are found in reports involving surface‐decoration of latex beads, albumin microparticles, or RBCs with Fg or Fg‐derived RGD peptides.^301^, ^302^, ^303^ Subsequently, several Fg‐decorated albumin microparticle constructs such as Synthocytes™, Thrombospheres™, and Fibrinoplate™, have been extensively evaluated as “synthetic platelet” technologies.^304^, ^305^, ^306^, ^307^ Maintaining consistent coating density and ensuring particle‐adhered fibrinogen stability are potential design challenges in this approach. Therefore, recent evolution of this approach has led to designs reported by the Lavik laboratory and the Olson laboratory, where instead of coating particles with the whole Fg protein, Fg‐relevant small molecular weight RGD peptides were utilized to decorate the surface of polymeric (PEG–PLA, PEG‐PLGA, polyurethane, etc.) nanoparticles.^308^, ^309^ These approaches have utilized linear small RGD peptide sequences, for example, CGRGD or GRGDS, that have binding capability to platelet surface integrin GPIIb‐IIIa, but these ubiquitous RGD sequences can also bind to many other integrins on different cells (i.e., lack of platelet‐specificity in vivo).^310^ These peptides can also reportedly trigger activation of resting platelets that could pose systemic thrombotic risk.^311^, ^312^, ^313^ Nevertheless, from a feasibility standpoint, decoration of micro‐ and nanoparticles with such RGD peptides has resulted in fibrinogen function‐mimetic constructs with promising hemostatic ability in vitro and in vivo.^308^, ^309^, ^314^ Platelet‐specificity of such designs can be potentially enhanced by utilizing peptides that have higher selectivity to the active platelet GPIIb‐IIIa. Examples of this are found in research reports on the utilization of Fg *γ*‐chain relevant H‐12 peptide, as well as our own research on using Fg function‐mimicking GPIIb‐IIIa‐specific cyclic RGD (cRGD) peptides (e.g., cyclo‐CNPRGDY[‐OEt]RC) to decorate albumin, polymeric, or liposomal particles with higher functional specificity in hemostatic action.^315^, ^316^, ^317^, ^318^, ^319^ From a mechanistic standpoint, all of these designs involving particle surface‐decoration with Fg or Fg‐derived peptides are essentially *super‐fibrinogen* systems that aim to amplify the aggregation of active platelets via multivalent interactions with platelet GPIIb‐IIIa integrins. Considering that intravenous delivery of human fibrinogen concentrate (e.g., RiaSTAP® by CSL Behring) is a clinically approved treatment for hypofibrinogenemia‐related bleeding complications, such *super‐fibrinogen* designs may find usage in unique clinical indications if successfully translated.

An additional capability for such peptide‐decorated particle platforms that mimic platelet adhesion or aggregation functions is their potential utilization as a drug delivery platform for injury site‐targeted delivery of bioactive drug cargo that can augment the hemostatic response. For example, the H‐12 peptide‐decorated liposomal particles have been used for targeted delivery of ADP (a platelet agonist) to enhance hemostatic outcomes in thrombocytopenia, trauma, and complex surgery animal models.^320^, ^321^, ^322^ In our research, we have demonstrated the utilization of cRGD peptide‐decorated liposomal particles for targeted delivery of Tranexamic Acid (TXA, an antifibrinolytic agent) to improve hemostatic outcome and survival in acute liver injury hyperfibrinolytic animal model.^323^ In another recent approach, we have demonstrated the feasibility of utilizing “VBP + CBP”‐decorated liposomal particles to encapsulate and deliver the enzyme thrombin (strong platelet agonist and converter of fibrinogen to fibrin) in an injury site‐selective manner to improve hemostatic outcome in tail‐clip bleeding and liver injury bleeding in coagulopathic animal models.^324^ These studies demonstrate the utilization of platelet‐inspired peptide‐decorated particle designs as effective drug delivery platforms for hemostatic agents that can supplement transfusion outcomes.

Physiologically, primary hemostasis is rendered by the cooperative action of the *adhesive* and *aggregatory* functionalities of platelets.^230^, ^325^ Therefore, “synthetic platelet” designs that can combine these mechanisms as either “mixture of particles” or on a single particle platform have garnered significant interest. For example, in one approach, latex beads surface‐decorated with rGPIb*α* protein fragments or with H‐12 peptides were mixed physically, or these two motifs were co‐decorated on a single bead, and in both cases the binding of particles on “VWF + collagen”‐coated surfaces was enhanced compared to particles bearing only one of the functionalities.^326^ Such studies also revealed that in combining two different motifs on a particle surface if there is a significant size difference between the motifs (e.g., large rGPIb*α* fragment compared to small H‐12 peptides), there may be mutual steric interference in their individual biointeractive ability. In our research, we have focused on potentially resolving such issues by using combinations of small peptides (11–20 amino acid residues) to cooperatively emulate the adhesive and aggregatory functions of platelets. For example, we have combined the adhesion‐promoting VBP and CBP peptides with the aggregation‐promoting cyclic RGD‐based Fg‐mimetic peptide (FMP) on liposomes or albumin‐based particles.^318^, ^327^, ^328^, ^329^, ^330^ These “functionally integrated” synthetic platelet systems could be scaled up, sterilized, and stored over a 6–9 month period of time, without compromising hemostatic bioactivity.^330^ These constructs showed higher hemostatic efficacy in vitro, as well as in vivo, compared to systems that had *adhesion only* or *aggregation only* mechanisms. The liposome‐based combination peptide decorated synthetic platelet design has been evaluated in several animal models of bleeding and coagulopathies, showing promising hemostatic performance.^328^, ^329^, ^330^, ^331^, ^332^, ^333^ These studies also demonstrate that such synthetic platelet systems can be species‐agnostic due to the absence of biological antigens (unlike platelet membrane‐coated systems), and thus can be developed for both veterinary and human applications. This technology was patented, registered as *SynthoPlate*, and licensed to Haima Therapeutics, to pursue translation and commercialization.

Recent advancements in this technology involve the utilization of cryoprotectant carbohydrates in the formulation to enable lyophilization into a powder that is expected to enhance shelf‐life and portability while preserving bioactivity. In an additional recent approach, we have also demonstrated that the combination of peptide‐decorated platelet‐inspired liposomal constructs can be further engineered to incorporate procoagulant thrombin‐amplifying anionic phospholipids (e.g., phosphatidylserine) that can remain masked by an enzyme‐cleavable PEG brush in circulation and be exposed at the injury site under the action of site‐specific enzymes (e.g., plasmin) to allow locally enhanced thrombin amplification and fibrin generation.^334^ This improved design demonstrated hemostatic rescue in scenarios where the performance of native platelets was severely compromised. Such approaches also demonstrate the potential for modularly manipulating the “ligand presentation properties,” “surface reaction properties,” and “encapsulated cargo properties” utilizing nanoscale biomaterials engineering to mimic and integrate various hemostatic mechanisms of platelets.

**FIGURE 8:**
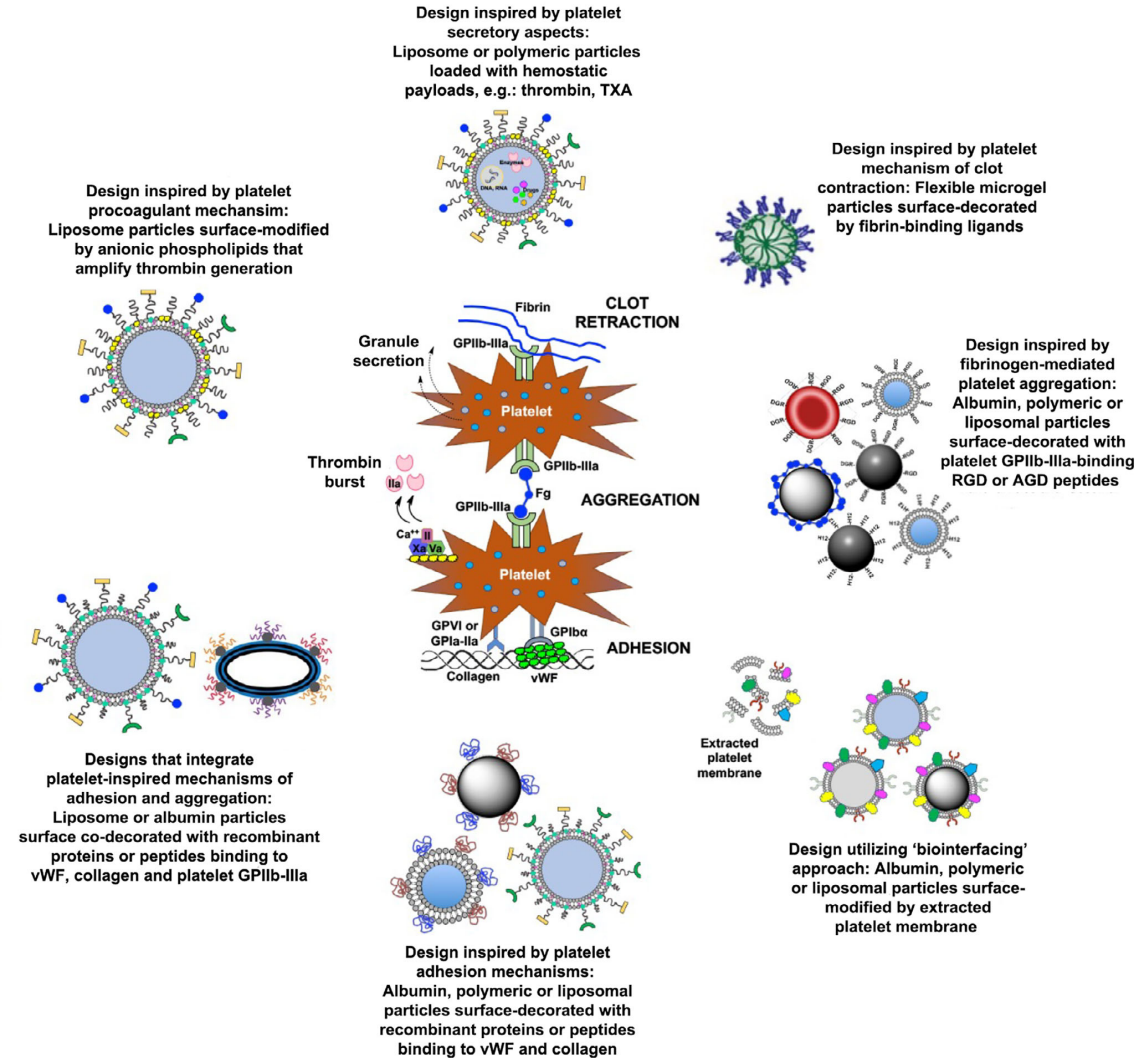
Representative designs of biomaterials‐based intravenous hemostatic technologies that mimic adhesive, aggregatory, procoagulant, or secretory properties of platelets.

Similar to scientific research emulating the morphological and mechanical properties of RBCs that was described previously, researchers have also explored the mimicry of platelet's biophysical aspects, for example, size, shape, and stiffness, which influence their fluid dynamic and margination behavior in vivo and thereby influence their hemostatic responses.^175^, ^176^, ^177^, ^178^, ^335^ The advent of unique bottom‐up and top‐down manufacturing methodologies to control particle geometry, morphology, and modulus has led to the potential incorporation of such *biophysical* design parameters into enhancing the performance of nanoparticle and microparticle constructs, including “synthetic platelet” systems. To this end, in collaboration with the Mitragotri laboratory, we have explored the platelet adhesion‐ and aggregation‐mimetic “VBP + CBP + FMP” *heteromultivalent* surface decoration on albumin‐based platelet‐shaped (discoid) particles that showed a higher biointeractive capability compared to spherical particles.^327^, ^336^ Related research in the Eniola‐Adefeso laboratory has also reported on the design of flexible microparticles for shuttling nanoparticles toward the vascular wall.^337^ In physiological hemostasis, stellate‐shaped activated platelets can also bind fibrin proto‐fibrils via the GPIIb‐IIIa integrins and pull on the fibrin to render clot contraction via the modulation of platelet cytoskeletal machinery, which is important for clot stability and wound healing.^338^ This unique biomechanical aspect of platelets was recently emulated in the work reported by the Brown and Barker laboratories, where polyisopropyl acrylamide‐based low‐crosslinked microgel platelet‐like particles (PLPs) were decorated with fibrin‐binding ligand motifs.^339^, ^340^ These flexible microgel particles demonstrated platelet‐relevant biomechanical clot contraction and hemostatic effect, with the requisite that the binding of these constructs needs prior presence of sufficient fibrin (i.e., significant coagulation output) at the injury site. Therefore, hematologic and coagulopathic conditions that present compromised fibrin generation (e.g., hemophilia, certain trauma‐associated coagulopathies, hypofibrinogenemia, etc.) may require additional refinement of this design for efficient hemostatic capability. On the other hand, in the presence of sufficient fibrin, this technology can not only bind to fibrin for platelet‐mimetic biomechanical clot contraction but can also act as a carrier platform for therapeutic cargo for clot‐targeted drug delivery.^341^, ^342^ Figure 8 depicts various design approaches for biomaterials‐based platelet surrogate technologies, and Table 3 provides a summary comparison of such designs in terms of materials, evaluation, and current status.

**TABLE 3:** Comparison summary of platelet‐derived and fully synthetic platelet surrogate systems.

Product	Material/Design	Mechanism	Preclinical evaluation	Preclinical outcome	Clinical trial No. and year	Primary clinical outcome	Current status
Cold‐stored Platelets (CSP or CS‐PLT)	Single donor‐derived platelets stored at 1–6°C	Induces platelet shape change, surface glycoprotein clustering and activation with enhanced procoagulant activity	Mouse, rabbit, rat and swine traumatic injury and thrombocytopenia models	Noninferior to transfusable platelets	NCT0249550, 2020 NCT0275441, 2018 NCT0378792, 2021 NCT0483441, 2025 NCT0614753, 2026	Superior to room temperature platelets for hemostasis in cardiac surgery and trauma, but with much shorter in vivo circulation time	FDA‐approved for clinical use when conventional room temperature platelets are not available
Lyophilized platelets (Thrombosomes, Cellphire Therapeutics)	Lyophilized pooled donor apheresis platelets	Partly conserves platelet aggregation, amplifies thrombin generation for fibrin formation	Rabbit, rat, guinea pig, dog and baboon bleeding and safety	Reduced blood loss, positive safety profile	NCT02223117, 2019 NCT04631211, 2023 NCT0577183, 2023	Positive safety profile in humans	Orphan drug designation by FDA for Acute Radiation Sickness
Cryopreserved Platelets (CPP, Cellphire Therapeutics)	Frozen pooled donor‐derived platelets	Increased phosphatidylserine expression for amplifying coagulation	Mouse traumatic hemorrhage, dog thrombocytopenia model	Improved clotting kinetics, improved MAP, reduced bleeding	NCT0475484, 2022 NCT0399148, 2025 NCT0470970, 2025	Noninferior to liquid stored platelets in surgical setting	Pending trial completion
Fibrinogen‐decorated particles (e.g., Synthocyte)	Albumin or polymer particles coated with fibrinogen	Promotes platelet aggregation	Rabbit ear bleed model, mouse thrombocytopenia model	Reduced blood loss, improved survival	N/A	N/A	Pre‐clinical
Recombinant Protein‐Decorated particles	Liposomes, polymer particles decorated with recombinant platelet‐relevant protein fragments	Mimics platelet adhesion to vWF and collagen	Rat bleeding model	Reduced blood loss	N/A	N/A	Pre‐clinical
Platelet membrane‐based systems (e.g., IPM by Cypress Bioscience)	Vesicles made from donor‐derived platelet membranes	Partly retains platelet membrane function, enabling adhesion and aggregation	Rat bleeding model	Partial retention of platelet bioactivity	Early‐phase trials explored for some systems	Unavailable	Pre‐clinical
Peptide‐decorated polymeric micro‐ and nanoparticles	Polymeric particles surface‐decorated with RGD peptides promoting platelet aggregatory functions	Enhances platelet aggregation via binding to platelet integrin GPIIb‐IIIa	Rat and pig bleeding models	Increased platelet aggregation and clotting outcomes	N/A	N/A	Pre‐clinical
SynthoPlate, (Haima Therapeutics)	Liposomal nanoparticles surface‐decorated with peptides mimicking platelet adhesive and aggregatory functions	Mimics platelet adhesion to vWF and collagen, Mimics fibrinogen‐mediated co‐aggregation of activated platelets	Mouse thrombocytopenia and trauma model, Rat, Rabbit and Swine hemorrhage model, Dog safety evaluation	Systemic safety with broad therapeutic window, Reduced blood loss, Improved survival in hemorrhage	N/A	N/A	Pre‐clinical
H12‐Liposomes and H12‐(ADP)‐liposomes	Liposomes decorated with fibrinogen‐relevant H12 peptide, loaded with ADP	Binds to activated platelets via GPIIb‐IIIa and locally releases ADP to promote more platelet activation	Rat and rabbit thrombocytopenic bleeding models, Rabbit cardiopulmonary bypass model	Enhanced platelet activation, reduced bleeding, rescued from coagulopathy	N/A	N/A	Pre‐clinical
Fibrin binding platelet like particles (PLP, Selsym Biotech)	pNIPMAM hydrogel particles functionalized with fibrin‐binding antibody fragment	Mimics fibrin‐adhesive and shape change mediated retraction properties of natural platelets	Mouse, rat, and swine bleeding models	Reduced blood loss, enhanced wound healing	N/A	N/A	Pre‐clinical
Thrombin‐sensitive fibrin‐binding platelet‐like particles (ts‐PLP, extension of PLP technology, Selsym Biotech)	pNIPMAM microgels crosslinked with thrombin‐cleavable peptides and conjugated to fibrin‐binding antibody fragment	Binds to fibrin, Responds to thrombin as a trigger for shape change to mimic platelet‐relevant clot retraction	Mouse bleeding model	Reduced blood loss, enhanced wound healing	N/A	N/A	Pre‐clinical

In addition to these approaches that are exploring the use of biophysical and biomechanical cues in particle‐based synthetic platelet design, some recent approaches have also reported biomaterials‐based *intravenous* hemostatic technologies that may be complementary to platelet and fibrin activities. For example, collaborative research between the laboratories of Stupp, Pritts, and Kibbe et al. have reported on intravenously injectable tissue factor (TF)‐targeted peptide amphiphile nanofibers that can localize to the vascular injury site to render bleeding control.^343^, ^344^ In another example, the Pun laboratory has reported on a linear water‐soluble (hydroxyethyl)methacrylate (HEMA) and N‐hydroxysuccinimide methacrylate (NHSMA) co‐polymer system [p(HEMA‐co‐NHSMA)] modified with a fibrin‐binding peptide, and intravenous delivery of this polymer, named polySTAT, showed the capability of crosslinking fibrin to emulate the role of coagulation factor FXIIIa in stabilizing fibrin.^345^, ^346^ In yet another example, the Mitragrotri laboratory adapted the VBP and CBP peptides to decorate hyaluronic acid (HA) polymer to create a system termed Hemostatic Agents via Polymer Peptide Interfusion (HAPPI) that, when intravenously delivered, could target the injury site and bind to exposed vWF and collagen for hemostatic action.^347^ In another recent interesting approach, researchers have reported on peptide‐based self‐assembled platelet‐like nanoparticles (pNPs) that bind to CD105 (endoglin) receptors on activated endothelium and transform morphologically into nanofibrous structures to form “artificial clots” that can have embolic effects on angiogenic blood vessels.^348^ Although this design has not been evaluated as an intravenous hemostat, it could have potential application in that area. One could envision combining such non‐particulate intravenous hemostatic agents with platelet‐mimetic particulate hemostat designs for augmented functions. Altogether, these studies present significant promise and opportunities to further investigate and optimize the hemostatic performance of synthetic platelet surrogates and platelet‐inspired intravenous hemostatic technologies via modularly mimicking and integrating a variety of platelet‐relevant biochemical as well as biophysical design parameters utilizing unique biomaterials tools.

## 4 BIOMATERIALS APPROACHES TO DESIGN WBC MIMICS

**FIGURE 9:**
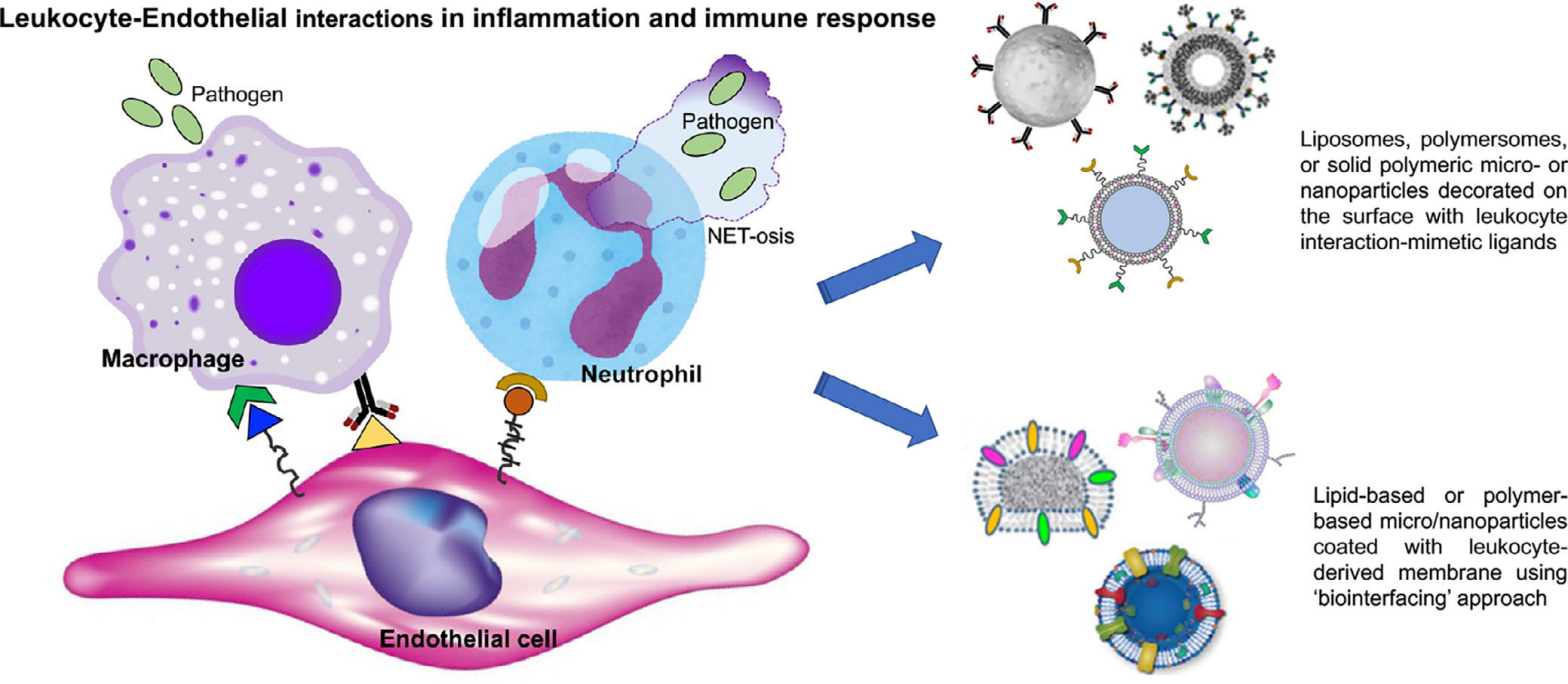
Schematic representation of leukocyte interactions with endothelium (e.g., via selectins, integrins, and other cell adhesion molecules) and design of “leukocyte surrogate” constructs that mimic such interactions via ligand decoration or membrane biointerfacing.

WBCs (e.g., neutrophils and macrophages) in blood maintain immune surveillance and protection against pathogens and diseased cells, and upon recognizing these targets, they render their cytotoxic destruction and phagocytic clearance via a complex concert of signaling, intercellular interactions, cytoplasmic granular secretions, and extracellular release of DNA (e.g., in neutrophil extracellular trap formation or NET‐osis). These complex processes are yet to be efficiently mimicked on synthetic particle platforms. However, some research studies have explored the design of WBC‐mimetic functions for enabling “targeted immune or inflammation modulatory response.” In one approach, poly (lactic acid)‐*co*‐poly (ethylene glycol), that is, PLA–PEG‐based biodegradable nanoparticles were surface‐decorated with monoclonal antibodies (mAbs) directed at receptors involved in leukocyte–endothelium interactions (e.g., E‐selectin, P‐selectin, VCAM‐1, and ICAM‐1), and these leukocyte‐inspired nanoparticle systems were found to undergo high binding to inflamed endothelium in vitro and in vivo.^349^ This design approach is similar to the leukocyte‐mimetic heteromultivalently ligand‐decorated polymer microparticles described previously that mimic leukocyte adhesion and rolling on inflamed endothelium.^292^, ^293^ In fact, the leukocyte‐inspired heteromultivalent ligand‐decoration approach was used in a design named “Leuko‐polymerosomes” where polymersome vesicles were surface‐decorated with selectin‐ and integrin‐binding motifs to render targeted interactions with “diseased” cells, and hydrogen peroxide (H_2_O_2_) was released from these vesicles to kill the target cells.^350^ In several subsequent designs, instead of utilizing antibody ligands to decorate synthetic particles, leukocyte‐derived membranes have been used to coat synthetic particles analogous to the RBC‐membrane and platelet‐membrane biointerfacing designs described previously. In one design named “Leukosomes,” membrane glycoproteins extracted from murine macrophages were reconstituted into proteoliposomes that could deliver drugs (e.g., Dexamethasone) to inflamed vasculature.^351^ Macrophage‐derived membrane has also been used to coat PLGA nanoparticles, and the resultant “macrophage decoy” constructs demonstrated the ability to sequester infection‐associated proinflammatory cytokines in a mouse model of sepsis, rendering promising therapeutic and survival benefit.^352^ In another design named “Leuko‐like Vectors,” nanoporous silica particles were surface‐coated with cellular membranes derived from leukocytes to render preferential binding to inflamed endothelium and transport therapeutics across the endothelium while avoiding lysosomal entrapment.^353^ In another approach, neutrophil‐membrane‐coated PLGA nanoparticles exhibiting the antigenic exterior and membrane functions of neutrophils were demonstrated to neutralize pro‐inflammatory pathways in an arthritis model in vitro and in vivo.^354^ Figure 9 shows a schematic of leukocyte interactions with the endothelium during immune response and inflammatory events, and the two current approaches in designing “leukocyte surrogate” systems. The membrane biointerfacing design approaches may present translational challenges regarding sufficient donor cell availability, scale‐up, and batch‐to‐batch compositional as well as functional reproducibility. Nonetheless, optimization of WBC‐mimetic particle designs utilizing surface ligand decorations or membrane biointerfacing, and their rigorous in vitro and in vivo evaluation for efficacy and safety, remain great research opportunities for the future.

## 5 PLASMA EXPANDERS, FREEZE‐DRIED PLASMA AND COAGULATION FACTOR CONCENTRATES

Heavy blood volume loss in acute hemorrhage scenarios necessitates rapid restoration of intravascular fluid volume to rescue blood pressure, body temperature, and oncotic balance, and this aspect has led to significant research and clinical use of *plasma expanders*.^355^, ^356^ Blood (hence plasma) volume loss results in the body trying to compensate for this deficit by redistribution of blood flow to vital organs (e.g., heart and brain), which in turn results in under‐perfusion of other tissues of the body, leading to multiple organ dysfunctions. An ideal plasma substitute/expander should be iso‐oncotic and isotonic with regard to natural plasma, have low viscosity and similar rheological properties as plasma, possess a reasonable half‐life of ~12 h, and show no adverse effects on the body while enabling hemodynamic restoration. To this end, two main classes of biomaterials that have been investigated as plasma substitutes/expanders in treating hypovolemia are crystalloids and colloids.^357^, ^358^ Crystalloids form crystals in their solid state and can dissolve in water as small molecules (e.g., sucrose, dextrose, etc.) or ions (e.g., sodium chloride, sodium bicarbonate, etc.) that can pass through a semi‐permeable membrane (e.g., cell membrane). The most well‐known crystalloids that have been used as *plasma surrogates* are 0.9% saline (sodium chloride), dextrose (5%–50% glucose in water), and Hartmann's solution or Ringer's lactate (1%–2% sodium bicarbonate). Colloids are fine particles that can form a dispersion or suspension in a continuous medium, and they tend to have higher circulation residence time than crystalloids since colloidal particles are too large to cross the vascular endothelium. Colloidal suspensions can also draw water in from the interstitial and intracellular fluid compartments because of their high osmolarity. The most well‐known biomaterial colloids that have been used as plasma surrogates are bio‐derived systems like human albumin suspensions (HAS) and cross‐linked gelatin suspensions (e.g., products like Gelofusine® and Haemaccel®), as well as semi‐synthetic polymers like hydroxyethyl starch (HES, e.g., the product Voluven®) and dextran. While all of the above crystalloids and colloids continue to be used in the clinic to various degrees, they have all shown some levels of side effects. For example, excess saline transfusion has shown effects of acidosis, hyperchloraemia, and impaired renal functions. Hartmann's solution shows detrimental effects in patients with impaired lactate metabolism and is also contraindicated in patients undergoing transfusion of citrate‐containing blood products to prevent the risk of coagulation. Excessive infusion of dextrose solutions can increase the risk of hyponatraemia, edema, acidosis, and thrombophlebitis. Among colloidal suspensions, albumin infusion has been associated with coagulopathic side effects due to the presence of vasoactive peptides. Infusion of gelatin suspensions has shown side effects on platelet functions and coagulation, possibly because gelatin is a derivative of collagen, which is a potent platelet activator. Dextran and other starch‐based plasma surrogate products have been implicated in anaphylactic reactions, as well as coagulopathy and impairment of renal functions. In spite of such side effects, these crystalloid and colloid‐based plasma surrogate/expander products continue to be used in clinical applications globally. Table 4 shows a summary comparison of the various plasma expander products that have undergone evaluation, with some being FDA‐approved. An emerging clinical approach in the treatment of traumatic hemorrhage and coagulopathy is that of *hypotensive resuscitation*. This approach avoids aggressive fluid resuscitation with colloid or crystalloid plasma expander systems and instead uses a low volume of resuscitative fluid to allow the patient to remain in a hypotensive state while adequately restoring organ perfusion and preventing hemodilution.^359^, ^360^ To this end, several unique polymeric systems are being studied to modulate the osmotic balance between tissue compartment and vascular compartment, and such polymeric systems, including PEG (20 K molecular weight), hyperbranched polyglycerol, and methacrylate‐based polymers, have shown promising results in preclinical animal models of bleeding.^361^, ^362^, ^363^, ^364^ Translational advancement of such polymeric systems will require rigorous preclinical and subsequent clinical evaluation. Instead of using plasma expanders and resuscitative fluids, actual isolated plasma from donors, if available, is of great benefit in transfusion medicine. Fresh donor plasma can be used for 1–5 days, and the storage (and availability) can be further enhanced by refrigeration (1–6°C), fresh‐freezing (FFP product stored at < −18°C), or converting into dry powder (e.g., freeze‐dried such as OctaplasLG® powder from Octapharma or spray‐dried such as FrontlineODP® from Velico). These plasma products are currently in clinical development. Certain plasma‐derived products like cryoprecipitate, prothrombin complex concentrate, and so forth, have also undergone clinical development and are currently approved for intravenous use in specific bleeding complications.

**TABLE 4:** Comparison summary of various plasma expander systems.

Product	Material/Design	Mechanism	Preclinical evaluation	Preclinical outcome	Clinical trial no. and year	Primary outcome	Current status
Hypertonic saline with dextran (HSD)	Colloidal, 7.5% NaCl, 6% Dextran‐70	Oncotic volume expansion	Porcine hemorrhage (liver injury)	Improved survival, increased MAP, pCO_2_ and plasma bicarbonate compared to normal saline	NCT00316017, 2009	No benefit to 28‐day survival in patients with hypovolemic shock	Terminated due to futility and safety concerns
Gelatin suspensions (Haemaccel®, Gelofusine®, Gelaspan®)	Colloidal, gelatin and solutes	Oncotic volume expansion	Controlled bleeding in Rats	Increased cardiac output and decreased viscosity compared to blood transfusion	Clinical trials done prior to year 2000 (NCT numbers not available)	No improvement to mortality or blood loss	Not approved by FDA
Hydroxyethyl starch (HES) (Hextend®, Voluven®)	Colloidal, 6% hydroxyethyl starch in Lactated Electrolyte Injection	Oncotic volume expansion	Porcine hemorrhage (liver injury)	Improved MAP and tissue oxygen saturation	NCT00983281, 2009	Reduced mortality, no iatrogenic coagulopathy	FDA‐approved
Human serum albumin	Colloidal, human albumin	Oncotic volume expansion	‐	‐	NCT00318942, 2012; SAFE trial, 2004	No improvement to mortality	FDA‐approved
PEGylated albumin	Human or bovine albumin, PEG	Increases capillary perfusion	Hamster controlled bleed	Improved survival	N/A	N/A	In early‐stage research
Ringer's lactate (RL)	Crystalloid, lactate	Volume expansion, replenishes electrolytes	Rat controlled bleed	Improved survival and mitigated acidosis in moderate shock; reduced survival and no effect on acidosis in severe shock	NCT04512950, 2020	No improvement to mortality or readmission	FDA‐approved

## 6 DISCUSSION

Transfusion of donor‐derived blood products is a clinical mainstay in the management of major surgeries, traumatic hemorrhage, and coagulopathies, hemorrhagic complications associated with childbirth, blood diseases such as anemia, sickle cell disease (SCD), and thalassemia, genetic bleeding disorders, transplant procedures, and chemotherapy or radiotherapy induced effects on bone marrow suppression impacting blood cell production. In all of these applications, there remains a persistent challenge of blood product availability stemming from the cumulative effects of donor dependency, perishable inventory, supply chain disruptions, and socio‐economic disparities. Synthetic blood surrogates can revolutionize transfusion medicine by potentially addressing these challenges associated with donor‐derived blood products, as well as the existing scaling and functional fidelity complications associated with bioreactor‐based in vitro blood cell production. Historically, the quest for a “blood substitute” is evident in reports regarding the use of milk, saline, Ringer's solution, or animal‐derived plasma during the 19th century.^365^, ^366^ While these materials might have functioned as volume replacement systems, they severely failed to render the biological functions such as oxygen transport and hemostasis that are performed by the natural blood cells. Through important discoveries and clinical studies during the past two decades, we have now come full circle to establish that human WB, if available, is the best transfusion product to mitigate bleeding complications and coagulopathy.^367^, ^368^, ^369^, ^370^, ^371^ If WB is not available, the next best choice is transfusion of isolated blood components (platelets, RBC, plasma) at controlled ratios. However, the utilization of WB or its components continues to face significant challenges stemming from donor shortage, special storage requirements, the need for rigorous hemovigilance and blood banking, and lack of widespread portability beyond major hospitals and large trauma centers. This is where synthetic blood surrogates can be envisioned to provide a potential bridge solution, by allowing donor‐independent large‐scale manufacture, sterilization, and long‐term storage as suspension or aqueous‐reconstitutable freeze‐dried powder, and on‐demand transfusion both within hospitals as well as in pre‐hospital settings (e.g., civilian emergency scenarios and austere battlefield conditions). Innovative transfusion logistics can also be envisioned by co‐formulating or co‐administering RBC surrogates and platelet surrogates along with plasma or coagulation factor concentrates, to constitute a biosynthetic *whole blood surrogate*. The possibility and promise of such approaches were recently demonstrated in studies where ADP‐loaded H‐12 peptide‐decorated liposomal particles were combined with HbVs in transfusion treatment of massive hemorrhage in thrombocytopenic rabbit models.^372^ In context, the United States Defense Advanced Research Project Agency (DARPA) recently announced a research program endeavor under their Biological Technology Office (BTO) directorate, named “Fieldable Solutions for Hemorrhage with bio‐Artificial Resuscitation Products” with a vision to engage our science, engineering, and clinical communities to team up and explore the possibility of creating a field‐deployable easily portable and storable biosynthetic blood surrogate.^373^ Biomaterials‐based technologies like HbV, Erythromer, SynthoPlate, PLP, PolySTAT, HAPPI, and so forth, have great potential to address such endeavors. These biosynthetic blood surrogate technologies can also be potentially integrated with plasma and plasma‐derived products to create unique resuscitative analogues for transfusion medicine. Biosynthetic blood surrogates can not only act as a bridge technology for transfusion logistics but can also allow conservation of natural blood products when the need becomes overwhelming (e.g., mass casualty scenarios).

The clinical translation of blood surrogate technologies will require careful navigation of many scientific, regulatory, and operational challenges. On the scientific side, beyond establishing efficacious and reproducible biologic functions that are close to the performance of natural blood products, biosynthetic systems can pose unwanted side‐effect risks including negative immune response, rapid clearance in vivo, off‐target effects, and narrow therapeutic windows.^374^, ^375^, ^376^, ^377^ Biomaterials approaches could potentially provide ways to address such challenges, as is evident from various circulation time enhancement and immunomodulatory approaches in biomedical areas of drug delivery and regenerative medicine. Additional scientific questions that will need to be addressed include determining circulation residence time of the biosynthetic blood cell‐mimetic components, establishing pharmacokinetic (PK) and pharmacodynamic (PD) profiles utilizing appropriate preclinical models, and investigating feasibility and risks associated with repeat administration and escalating dose to determine maximum tolerated dose and therapeutic window. On the manufacturing side, establishment of biosynthetic blood surrogate technologies will require establishment of cost‐effective and efficient large‐scale GMP manufacturing processes, standardized in vitro characterization metrics, and standardized in vivo evaluation for performance metrics in rigorous preclinical models to establish safety and efficacy profiles for investigational new drug (IND) applications. On the regulatory side, translation of biosynthetic blood products will require rigorous communication with the FDA to establish appropriate categorization of the products (e.g., drug vs. biologic) and thereby establish the appropriate pathway for clinical studies to demonstrate safety and efficacy in patients. It is important to note here that Hb‐based biosynthetic RBC surrogates may be considered a *biologic* product due to the presence of biologically sourced Hb, while fully synthetic systems like PFCs as O_2_‐carriers, particulate systems, and polymeric systems as platelet‐inspired hemostats, etc., may be categorized as a *drug* product. These scientific and translational milestones can be time‐consuming and resource‐intensive and will require significant interdisciplinary and inter‐institutional efforts.

## 7 AN OPPORTUNITY FOR GLOBAL HEALTH EQUITY IN TRANSFUSION MEDICINE

According to the WHO, a “blood product” is an essential therapeutic substance to support human health, improve survival probability, and quality of life.

**FIGURE 10:**
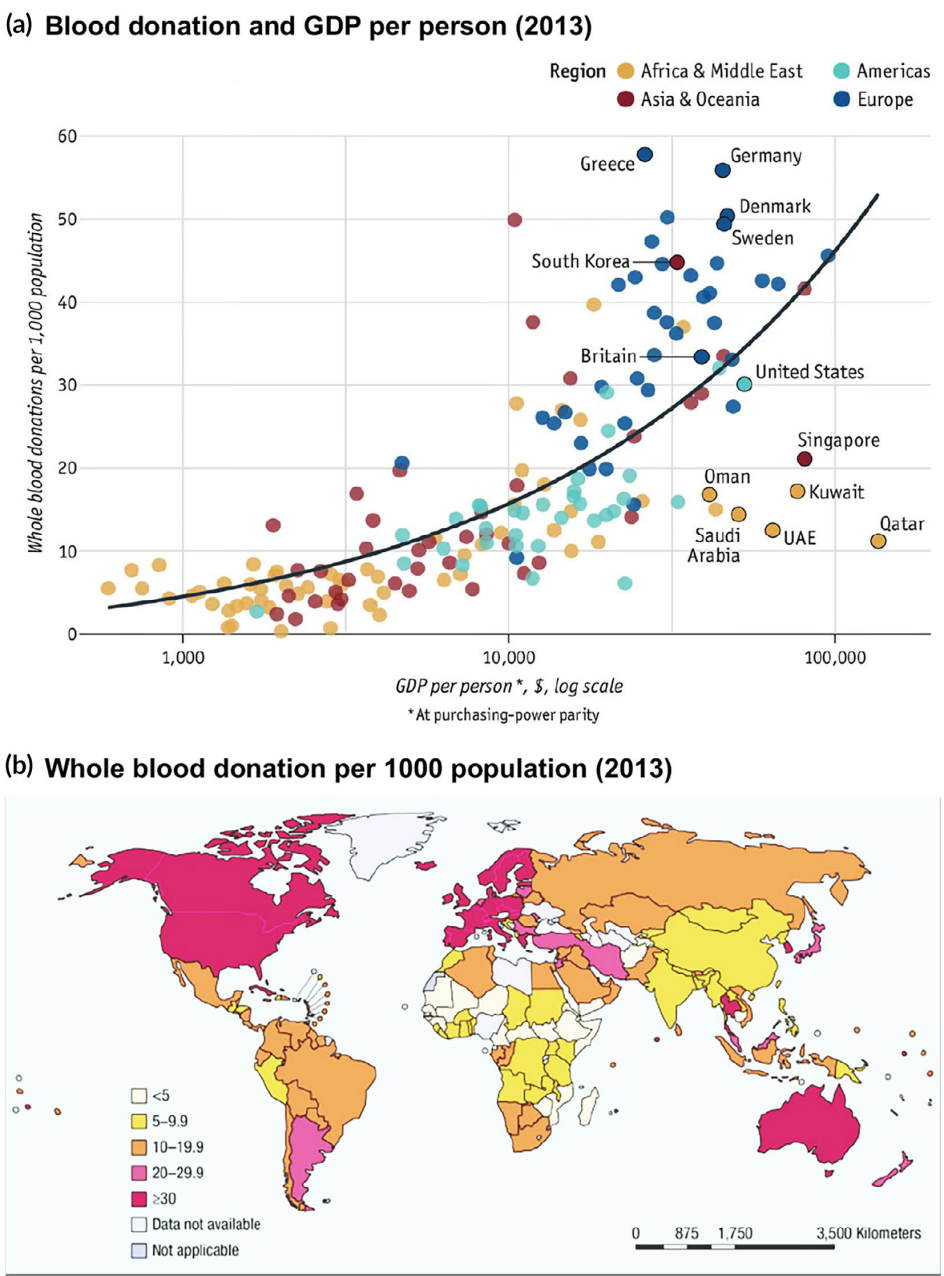
Disparity in blood donation at a global scale across high‐income, middle‐income, and low‐income regions (adapted from Global status report on blood safety and availability 2016. Geneva: World Health Organization; 2017. License: CC BY‐NC‐SA 3.0 IGO.)

However, donor‐derived blood collection, testing, storage, distribution, and utilization are highly disparate among various parts of the world (Figure 10), with high‐resource regions having significantly better hemovigilance and donor management than low‐resource regions.^378^, ^379^ This poses a formidable health hazard of transfusion‐transmitted infection risks from poor hemovigilance in low‐income regions of the world. Furthermore, in a global framework, there are often major phenotypic differences between the more well‐monitored donor pool and the actual patient population where such donor blood is largely used. For example, SCD is an inherited hemoglobinopathy with a major number of patients being of African descent, and these patients require periodic blood transfusions.^380^ However, a large part of the blood donor population is of Western descent, who have a different Rh profile than the African population. This can lead to phenotypic and genotypic challenges. Therefore, diversifying the donor population is essential to meet such demographic needs, which is an additional challenge considering that the overall donor availability is already limited. Furthermore, there are specific populations in the world who choose not to receive blood transfusions due to their religious beliefs, for example, Jehovah's Witnesses.^381^ These various challenges present an opportunity to address health equity in transfusion medicine via the development of biomaterials‐based biosynthetic blood surrogates. Because of the synthetic nature of the products, such blood surrogate technologies will potentially not require type matching and can be used universally. Also, with large‐scale GMP manufacturing and potential lyophilization, such products could be transported and stored for long times in various climate conditions and made available on demand to meet the various global needs. With the continued evolution of advanced manufacturing processes, improved storage and distribution logistics, and refinement of transfusion strategies, one can envision a future where *biosynthetic blood surrogates* can become complementary or adjunctive to *natural blood products* in the toolbox of transfusion medicine, to allow timely and equitable use in the global population. Considering the fact that biomaterial‐based nanoparticulate therapeutic formulations (including recent Moderna, Pfizer, and Astra‐Zeneca vaccines for COVID‐19) continue to be translated globally into clinical use, it can be envisioned that biomaterials and nanomedicine strategies could potentially achieve safe and effective biosynthetic technologies for blood surrogate applications.^133^, ^382^, ^383^, ^384^ The biomaterials and nanobiotechnology fields have a great opportunity to make critical and significant contributions in this field and continue to be the drivers of important biomedical innovations for the future.

## AUTHOR CONTRIBUTIONS

**Dante Disharoon:** Conceptualization, Collection and analysis of data and information, Writing original draft sections, Final preparation of Tables, Review and editing of final manuscript. **Sonali Rohiwal:** Conceptualization, Collection and analysis of data and information, Writing original draft sections, Final preparation of Tables, Review and editing of final manuscript. **Selvin Hernandez:** Preparation of draft Tables, review and editing of final manuscript. **Bipin Chakravarthy Paruchuri:** Preparation of draft Tables and Figure Components. **Rohini Sekar:** Preparation of draft Tables and Figure Components. **Norman F. Luc:** Writing original draft sections, reviewing and editing of final manuscript. **Anirban Sen Gupta:** Conceptualization, Supervision, Funding acquisition, Writing original draft sections, Review and editing of final manuscript, Manuscript submission.

## CONFLICT OF INTEREST STATEMENT

ASG is an inventor on patents involving the composition and use of Synthetic Platelets (US 9107845, US 9636383, US 10426820, US 10434149). He is also a co‐founder of Haima Therapeutics, a biotech start‐up company focused on the research and development of blood surrogate technologies where the above patents are licensed. ASG serves as the Chief Technology Officer of Haima and the chair of Haima's Scientific Advisory Board (SAB). DD, SR, SH, BCP, and NFL do not have any conflict of interest.

## Data Availability

This is a comprehensive review paper on biosynthetic approaches to blood surrogate technologies. All data and information presented here are available in appropriate references stated in the manuscript text and, where applicable, in figure captions.
